# Synthesis, Cytotoxicity, and Mechanistic Evaluation of Tetrahydrocurcumin-Amino Acid Conjugates as LAT1-Targeting Anticancer Agents in C6 Glioma Cells

**DOI:** 10.3390/ijms252011266

**Published:** 2024-10-19

**Authors:** Polsak Teerawonganan, Hasriadi, Peththa Wadu Dasuni Wasana, Pornpoom Angsuwattana, Apichart Suksamrarn, Nonthaneth Nalinratana, Opa Vajragupta, Pasarapa Towiwat, Worathat Thitikornpong, Pornchai Rojsitthisak

**Affiliations:** 1 Center of Excellence in Natural Products for Ageing and Chronic Diseases, Chulalongkorn University, Bangkok 10330, Thailand; nuttpolsak@gmail.com (P.T.); adhiehasri@gmail.com (H.); nonthaneth.n@chula.ac.th (N.N.); opa.v@chula.ac.th (O.V.); pasarapa.c@chula.ac.th (P.T.); worathat.t@pharm.chula.ac.th (W.T.); 2 Biomedicinal Chemistry Program, Department of Biochemistry and Microbiology, Faculty of Pharmaceutical Sciences, Chulalongkorn University, Bangkok 10330, Thailand; 3 Department of Pharmacology and Physiology, Faculty of Pharmaceutical Sciences, Chulalongkorn University, Bangkok 10330, Thailand; 4 Department of Pharmacy, Faculty of Allied Health Sciences, University of Ruhuna, Galle 80000, Sri Lanka; dasuniwasana@ahs.ruh.ac.lk; 5 Department of Food and Pharmaceutical Chemistry, Faculty of Pharmaceutical Sciences, Chulalongkorn University, Bangkok 10330, Thailand; 6136540333@student.chula.ac.th; 6 Department of Chemistry and Center of Excellence for Innovation in Chemistry, Faculty of Science, Ramkhamhaeng University, Bangkok 10240, Thailand; asuksamrarn@yahoo.com; 7 Molecular Probes for Imaging Research Network, Faculty of Pharmaceutical Sciences, Chulalongkorn University, Bangkok 10330, Thailand

**Keywords:** tetrahydrocurcumin, curcumin, glioblastoma, cancer, amino acid transporter, LAT1

## Abstract

Glioblastoma, a fatal brain cancer with limited treatments and poor prognosis, could benefit from targeting the L-type amino acid transporter I (LAT1). LAT1 is essential for cancer cells to acquire necessary amino acids. Tetrahydrocurcumin (THC), a key curcumin derivative, shows potential for glioblastoma treatment. However, its effectiveness is hindered by poor physicochemical and pharmacokinetic properties. Therefore, this study aims to improve the therapeutic efficacy of THC against glioblastoma by chemically modifying it to target LAT1. A novel series of THC-amino acid conjugates were synthesized by conjugating five amino acids: glycine, leucine, isoleucine, and phenylalanine to THC via carbamate bonds. The therapeutic efficacy of THC-amino acid conjugates was further examined in C6 glioma cells, including the role of LAT1 in their therapeutic effects. Among the conjugates tested, THC conjugated with two phenylalanines (THC-di-Phe) showed remarkably higher cytotoxicity against C6 glioma cells (35.8 μM) compared to THC alone (110.7 μM). THC-di-Phe induced cellular death via necrosis and apoptosis, outperforming THC. Additionally, THC-di-Phe inhibited C6 cell proliferation and migration more effectively than THC. Co-incubation of THC-di-Phe with the LAT1 inhibitor 2-Aminobicyclo-(2,2,1)-heptane-2-carboxylic acid (BCH) further increased cellular death. THC-di-Phe also significantly inhibited the P70SK/S6 pathway, regulated by LAT1 inhibitors, more effectively than THC and displayed a similar binding mode with both JX-075 and BCH to the active site of LAT1. Findings suggest the potential role of THC-di-Phe as a LAT1 inhibitor and provide novel insight into its use as a potent antitumor agent in glioma with increased therapeutic efficacy.

## 1. Introduction

Glioma is a primary and aggressive tumor in the central nervous system (CNS) with a poor clinical prognosis. The most common type of glioma is glioblastoma, which has about 61.5% incidence [1]. Numerous pharmacological and non-pharmacological approaches have been applied to treat glioma, including surgery, radiotherapy, chemotherapy, and combinations [2]. The standard treatment for glioma is surgery, followed by the treatment of radiotherapy and adjuvant chemotherapeutic drugs, such as temozolomide [3]. Although this approach seems effective in reducing tumor progression, the efficacy still fails to extend the median survival of patients. Patients with glioblastoma multiforme have a less than 3% survival rate at three years. The optimum treatment for glioblastoma patients only contributes to a median survival rate of ≤1% [4]. In addition, the use of chemotherapeutic drugs results in severe side effects, such as gastrointestinal disturbances, liver toxicity, vomiting, and nausea [5]. Therefore, finding chemotherapeutic drugs with better efficacy and safety profiles is urgently required to combat glioma.

Tetrahydrocurcumin (THC), a major metabolite of curcumin, has the potential use in treating CNS diseases, including Alzheimer’s disease [6,7], neuroinflammation [8,9], Parkinson’s disease [10], traumatic brain injury [11], ischemic stroke [12], and brain cancer [13]. This versatility underscores the potential significance of THC in addressing the complex challenges associated with CNS disorders, including glioma [14]. THC interreacted synergistically with radiotherapy and effectively inhibited the growth of brain cancer in C6 cells by suppressing the expressions of cyclin D1 and proliferating cell nuclear antigens [13]. In addition, nanoformulation of THC combined with doxorubicin and radiotherapy demonstrated better profiles in suppressing cancer behaviors in C6 glioma cells [15]. Though several biological and pharmacological potentials of THC have been reported, its efficacy is still minor and hindered by its poor physicochemical and pharmacokinetic profiles. THC elicits low aqueous solubility and low intestinal absorption, limiting its ability to reach systemic circulation [16].

Nanoparticle-based delivery systems [17], intranasal drug delivery [18], chemical modification [19], and p-glycoprotein inhibition [20] are key strategies to enhance brain drug delivery. Among these, chemical modification is a widely used approach to overcome the limitations associated with active compounds, such as THC [21,22]. One effective method involves conjugating active compounds with amino acids. Several active compounds conjugated with various amino acids exhibited better efficacy than their parent molecules, such as valproic acid [23], gemcitabine [24], dopamine [25], ferulic acid [26], vortioxetine derivatives [27], and curcumin [28]. The advantages of amino acid conjugates include improving the stability of active compounds, being safe, and providing additional efficacy [29]. Specifically, the use of amino acids in drug development for targeting CNS diseases has been widely used, including β-alanine-derived sulfamides containing L-valine and L-phenyl alanine skeletons, ester L-phenylalanine amide and ester conjugates, XP13512, N-Gly-CBZ, N-cysteamine carbamazepine, LY544344, and lisdexamfetamine dimesylate [30]. The presence of amino acid structures improves pharmacokinetic profiles and the therapeutic efficacy of active compounds. The presence of a high number of amino acid transporters in the blood–brain barrier (BBB), such as L-type amino acid transporters 1 (LAT1) and 2 (LAT2), improves the delivery of amino acid conjugates to the CNS [31,32]. Furthermore, abundant amino acid transporters in central immune cells, including the microglia and astrocytes, make it more efficacious once it reaches the brain because the transporter facilitates the uptake of the parent drug into the microglia and astrocytes [32].

LAT1 is an amino acid transporter highly expressed in cancer cells, particularly brain cancers. Physiologically, LAT1 functions as a transporter of essential amino acids to the cells, such as cysteine, histidine, glutamine, isoleucine, leucine, methionine, phenylalanine, tryptophan, and valine [31,33]. The expression of this transporter in cancer cells is associated with poor prognosis, increased tumor growth, tumor migration, and angiogenesis [34]. Hence, LAT1 has been targeted for cancer diagnosis, and delivering active compounds and suppressing LAT1 activity is considered a treatment option for cancer [35]. In line with that, several amino acid conjugates for targeting LAT1 have been developed. The valproic acid-phenylalanine, lysine-ketoprofen, phenylalanine-ketoprofen, phenylglycine-mustard, and melphalan are amino acid conjugates developed as substrates of LAT1, in which their conjugation with amino acids improved delivery of active agents through LAT1. In addition, several non-transportable compounds of drugs targeting LAT1 have been developed, and some of them are being tested in clinical trials, such as BCH (an α-amino acid with a hydrophobic bulky side chain), JPH203 (a triiodothyronine-derived amino acid), and bicyclic *meta*-tyrosine-based LAT1 inhibitors including JX-009, JX-075, JX-078, JX-119, and SKN101 [35]. These are anticancer agents with amino acid promoiety that suppress the LAT1 activity. The suppression of LAT1 contributes to amino acid deprivation and inhibition of the mTOR/p70S6/S6 pathway, leading to decreased tumor proliferation and progression [33,35].

In line with the previous literature supporting improved therapeutic efficacy of parent drugs by their amino acid conjugates, a series of THC amino acid conjugates were synthesized in the present study to reduce the dose requirement of THC. The synthesized THC conjugates were tested in C6 glioma cells, and the involvement of the LAT1 in the therapeutic effects of THC amino acid conjugates was also evaluated.

## 2. Results and Discussion

### 2.1. Synthesis

In this study, four amino acids were selected: glycine, leucine, isoleucine, and phenylalanine, all of which are substrates for LAT1, except for glycine, which is used as a negative control. The amino acids were conjugated to THC via a carbamate bond to achieve a novel series of THC-amino acid conjugates. These THC-amino acid conjugates are expected to enhance the anticancer properties of THC by modulating LAT1. The conventional procedure for carbamate ester bond formation typically involves two stages. Initially, the primary amine reacts with phosgene to produce a reactive isocyanate derivative. In the following step, the intermediate is reacted with the hydroxyl group of the reactants [36]. The phosgene used in this process is highly harmful to pulmonary alveoli and has a poor yield, so its use in carbamate linker production is not recommended. Consequently, an activated 4-nitrophenyl carbamate intermediate was used to produce carbamate bonds [37].

**Figure 1:**
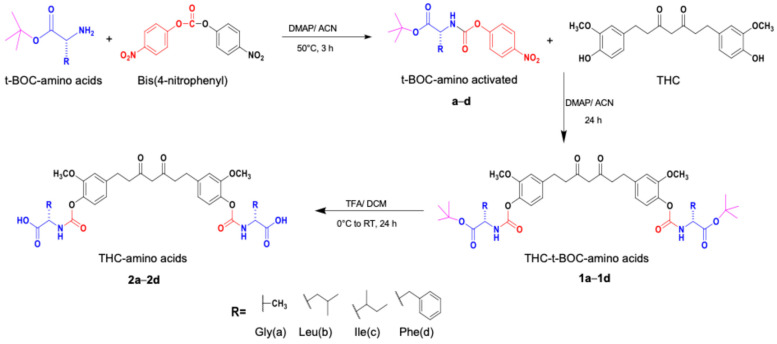
Synthesis routes of THC-amino acid carbamate ester conjugates: THC-di-Gly, THC-di-Leu, THC-di-Ile, and THC-di-Phe.

Briefly, THC-amino acid carbamate ester conjugates were synthesized in three steps, as shown in Figure 1. In the first step, the amino group of each *C*-protected amino acid was reacted with bis(4-nitrophenyl) carbonate in the presence of 4-dimethylaminopyridine (DMAP) as a catalyst to obtain activated 4-nitrophenyl carbamate intermediate with good yields, 75–82%. Next, the activated intermediates were conjugated with the THC to obtain the THC-*t*-Boc-amino acid carbamate esters with 65–67% yields. The nitrophenol group of the active intermediate was substituted by the nucleophile, a hydroxyl group of THC, resulting in the carbamate function, a linker between THC and an amino acid. The proton NMR results indicated that the t-Boc-group exhibited a characteristic -CH_3_ chemical shift between 1.4 and 1.5, corresponding to 18 protons of tertiary methyl groups of two *t*-Boc promoieties, demonstrating its conjugation at both phenolic -OH positions of THC. In the last step, THC-*t*-Boc-amino acid carbamate ester conjugates were deprotected using trifluoroacetic acid (TFA) in dichloromethane. All the final compounds were purified by flash chromatography and obtained with good yields: Tetrahydrocurcumin-di-Glycine (THC-di-Gly, 75% yield), Tetrahydrocurcumin-di-Leucine (THC-di-Leu, 90% yield), Tetrahydrocurcumin-di-Isoleucine (THC-di-Ile, 85% yield), and Tetrahydrocurcumin-di-Phenylalanine (THC-di-Phe, 77% yield). The structures of all synthesized compounds were further confirmed by proton and carbon NMR spectroscopy and HRMS spectroscopy (Supplementary Figures S1–S28 and Tables S1–S7). The structural elucidation results agreed with the assigned structures in all cases (Table 1).

### 2.2. Cytotoxicity Profiles of THC-Amino Acid Conjugates in C6 Glioma Cells and BJ Fibroblast Cells

**Figure 2:**
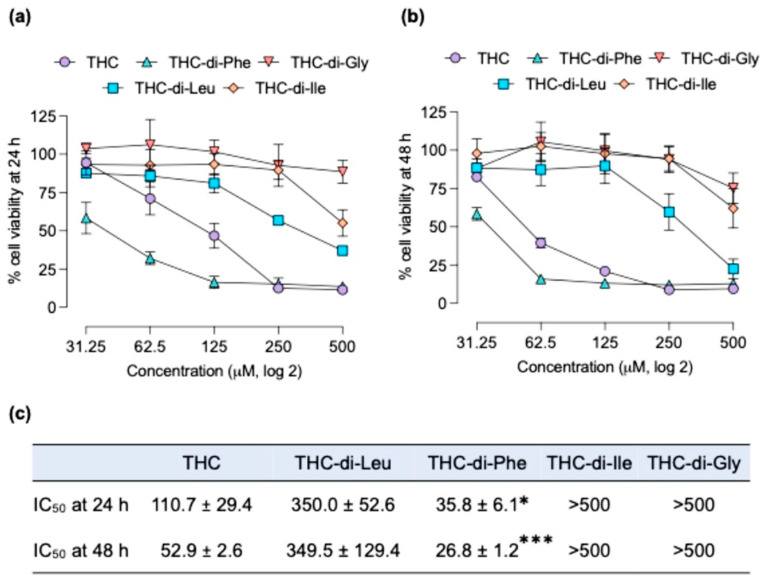
Cytotoxicity profiles of THC-amino acid conjugates in C6 glioma cells. The cytotoxicity was assessed at (**a**) 24 h and (**b**) 48 h post-treatment. (**c**) The degree of cytotoxicity between THC and its amino acid conjugates was further analyzed using their respective IC_50_ values using non-linear regression. n = 3 independent experiments. The IC_50_ values were compared using the student *t*-test. * *p* < 0.05 and *** *p* < 0.001 compared to the THC-treated cells.

The cytotoxicity profiles of THC-amino acid conjugates in C6 glioma cells were assessed for 24 and 48 h post-compound treatments. After 24 and 48 h, the cell viability assay was performed, and the IC_50_ of each compound was determined. The results showed that THC-di-Phe demonstrated strong induction of cell death compared to that of its parent drug, THC, while other THC-amino acid conjugates failed to enhance the anticancer efficacy of THC. The IC_50_ of THC and THC-di-Phe at 24 h was 110.7 and 35.8 µM, respectively, whereas the IC_50_ at 48 h was 52.9 and 26.8 µM, respectively. Only THC-di-Phe demonstrated promising activity in inducing cell death in the C6 cells compared to other THC-amino acid conjugates (Figure 2a–c). This could be due to the higher affinity of phenylalanine to LAT1 than the other amino acids [38]. Glycine, which was used as a negative control, does not possess stereoisomers, unlike other amino acids that exist in L- and D-forms. As LAT1 primarily transports L-isomers, this could explain the lack of enhancement in cytotoxicity observed with THC-di-Gly conjugates. Moreover, the observed IC_50_ value for THC was consistent with previous studies wherein the IC_50_ of THC is substantially higher than traditional chemotherapeutic agents [13,39]. Conjugating THC with diphenylalanine significantly improved its cytotoxic effects in glioma cells, indicating a novel mechanism of action and pharmacological advantages over the parent compound.

Furthermore, these enhanced therapeutic effects not only benefit the efficacy of THC as a single compound but also demonstrate promising implications for its therapeutic application in cancer. Previous research has established THC’s potential to interact with other anticancer drugs, resulting in synergistic anticancer effects [40]. Additionally, THC has been shown to enhance the therapeutic potential of radiation therapy against glioma [13]. Therefore, THC-di-Phe could represent a promising candidate for inclusion in optimal therapeutic regimens for the treatment of glioma.

After determining the IC_50_, THC and THC-di-Phe were tested at 5 to 40 µM to assess non-toxic concentrations. Results showed that THC-di-Phe concentrations above 10 µM were toxic to C6 glioma cells (Supplementary Figure S29). These results were further applied in cell immigration, proliferation, and immunoblotting assays.

**Figure 3:**
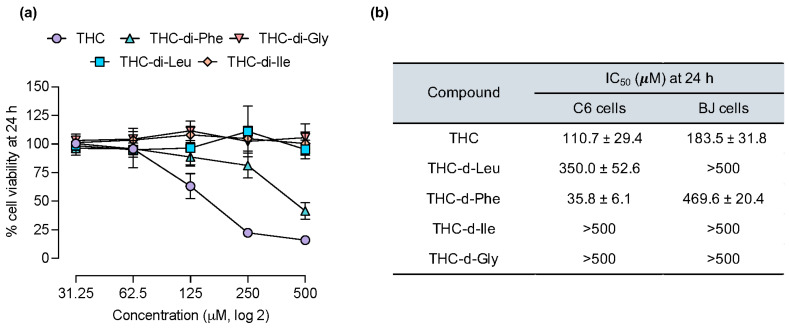
Cytotoxicity of THC-amino acid conjugates in BJ fibroblast cells. (**a**) Cytotoxicity profiles at 24 h. (**b**) Comparison of cytotoxicity in C6 glioma cells and BJ fibroblast cells. Data are presented as mean ± SD from three independent experiments.

To evaluate the selectivity of the test compounds, their cytotoxicity against normal BJ fibroblast cells was assessed. As shown in Figure 3a,b, THC and THC-d-Phe exhibited IC_50_ values of 183.5 ± 31.8 and 469.6 ± 20.4 µM, respectively, in BJ cells. All other compounds showed IC_50_ values above 500 µM. Figure 3b further highlights the selectivity of THC-d-Phe. In C6 glioma cells, THC exhibited lower cytotoxicity compared to THC-d-Phe, while in BJ cells, THC was more toxic than THC-d-Phe. Additionally, the cytotoxicity of THC-d-Phe was significantly higher in C6 glioma cells than BJ fibroblast cells. These results indicate that THC-d-Phe demonstrates greater toxicity in cancer cells with minimal cytotoxic effects on normal cells, suggesting its potential for selective action against cancer cells.

### 2.3. Apoptosis and Necrotic Profiles of C6 Glioma Cells Induced by THC-di-Phe

**Figure 4:**
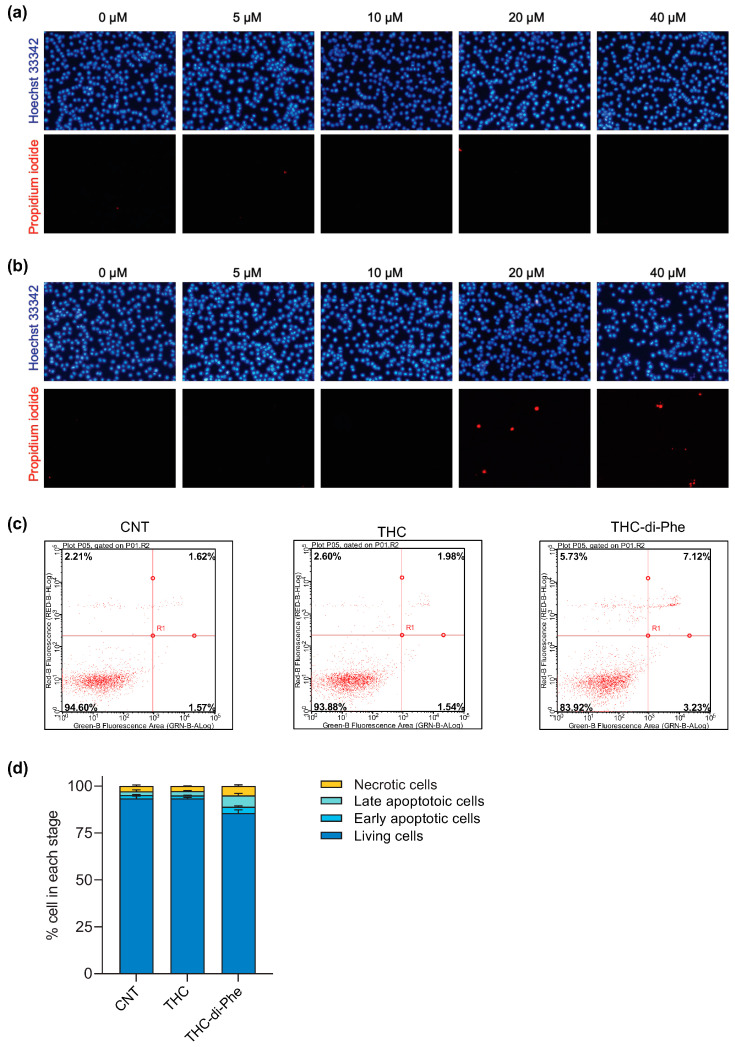
Assessment of apoptosis and necrosis in C6 glioblastoma cells after treatment with THC and THC-di-Phe. (**a**,**b**) The cells are treated with CNT (0.5% dimethyl sulfoxide, DMSO), THC (**a**), and THC-di-Phe (**b**) at 5, 10, 20, and 40 µM concentrations, followed by the Hoechst 33342/PI double staining assay. (**c**,**d**) Annexin-V/PI analysis by flow cytometry in C6 cells. The cells are treated with CNT (0.5% DMSO), 20 µM THC, and 20 µM THC-di-Phe, followed by cellular necrosis and apoptosis assessments by flow cytometry. (**c**) Representative dot plots of Annexin V/PI staining flow cytometry data for the CNT, THC, and THC-di-Phe. (**d**) Percentage of cells in the stages of necrosis, early apoptosis, and late apoptosis. Data are presented as means ± S.D. (n = 3).

Followed by the substantial findings in the cytotoxicity profile of THC-di-Phe, the mode of cellular death was further analyzed by the Hoechst 33342/propidium iodide (PI) double staining assay. As shown in Figure 4a,b, higher THC-di-Phe concentrations resulted in fewer cells covering the area than its equimolar concentrations of THC. THC-di-Phe showed high apoptotic profiles, indicated by increased intensity of blue color and the presence of fragmented, blue-stained cellular components. Induction of cellular apoptosis by THC-di-Phe was observed at 20 and 40 µM equimolar to THC and higher concentrations. Furthermore, a higher amount of necrosis was observed in the cells treated with THC-di-Phe compared to that of THC, as indicated by red fluorescence after PI staining, which is in line with the apoptosis induction. The results validate better cytotoxicity profiles of THC-di-Phe compared to THC and demonstrate that the mode of cellular death is induced via necrosis and apoptosis.

### 2.4. Annexin V/PI Double Staining Assay by Flow Cytometry

The comparison of the effects of THC and THC-di-Phe on cell death stages was analyzed using flow cytometry. As shown in Figure 4c, control cells demonstrated normal cell viability without significant apoptosis or necrosis. THC-di-Phe possessed higher induction of cell death than CNT- and THC-treated cells. Specifically, when C6 glioma cells were treated with 20 μM of THC-di-Phe, the percentage of late apoptotic cells significantly raised to 6.07 ± 1.13%, in contrast with control or THC-treated cells (2.06 ± 0.69% and 2.33 ± 0.32%, respectively). Moreover, THC-di-Phe induced higher necrotic cell death, 5.01 ± 0.64%, compared to control and THC groups, 2.76 ± 0.48 and 2.73 ± 0.15, respectively (Figure 4d). Overall, the results indicate the induction of cell death via apoptosis and necrosis in cancer cells by THC, in which the apoptotic induction ability of THC-di-Phe is significantly higher than THC.

### 2.5. The Effects of THC-di-Phe on Cell Proliferation in C6 Cells

**Figure 5:**
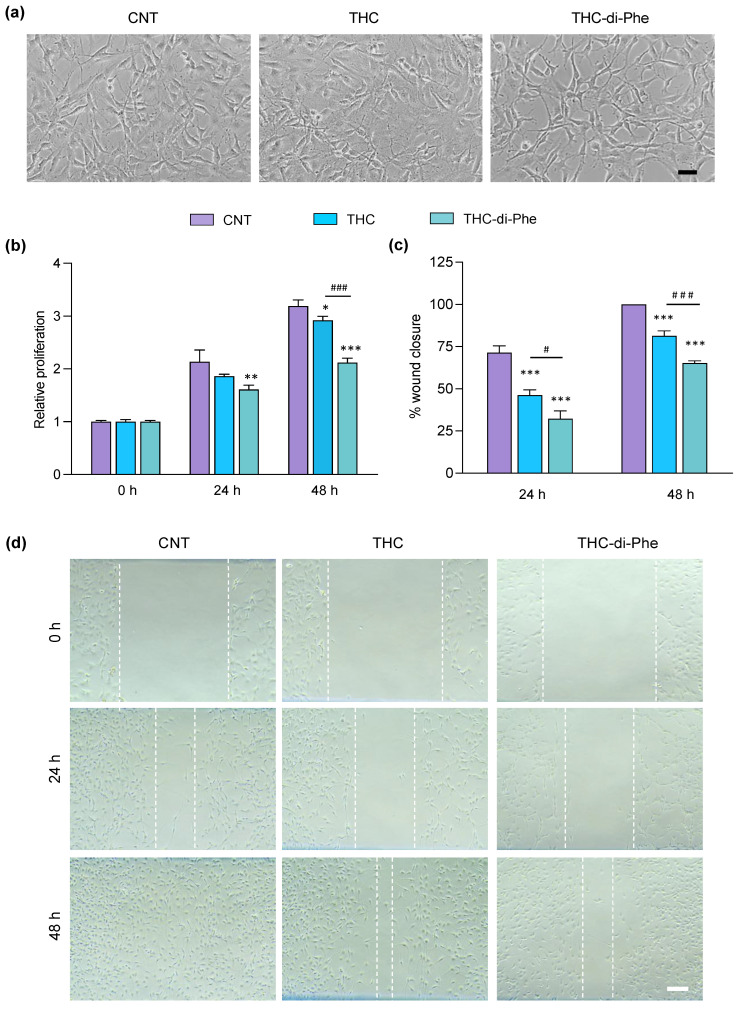
Effect of THC-di-Phe on glioma cell proliferation and migration. (**a**,**b**) Antiproliferative effect of THC and THC-di-Phe on C6 glioma cells. (**a**) Representative images (phase contrast) of cell proliferation assay at 48 h post-treatment. Scale bar: 30 µm. (**b**) The relative proliferation of cells treated with THC and THC-di-Phe at 24 and 48 h post-treatments (n = 3 independent experiments). (**c**,**d**) Effect of THC-di-Phe on glioma cell migration. (**c**) The migration rate of the wounded region presented as % of wound closure compared to 0 h (n = 3 independent experiments). (**d**) Cell migration images of C6 glioma cells at 0, 24, and 48 h after monolayer wounding. Scale bar: 10 µm. The cells were treated with vehicle (0.5% DMSO), 10 µM THC, and 10 µM THC-di-Phe, followed by assessment of cell viability and wound width at 0, 24, and 48 h post-treatment. Data are presented as mean ± SD; * *p* < 0.05, ** *p* < 0.01, *** *p* < 0.001 vs. CNT, ^#^ *p* < 0.05, ^###^ *p* < 0.001 vs. THC.

The ability of THC to suppress C6 glioma cell proliferation has previously been reported [13]. Not only in glioma cells, but inhibition of cell proliferation by THC was also observed in other types of cancer cells, including non-small cell lung carcinoma cells [41], human leukemia HL-60 cells [42], and human breast cancer cells [43]. Hence, the antiproliferative effect of THC-di-Phe was assessed using a cell proliferation assay to determine whether the conjugation with amino acid Phe could increase the efficacy of THC. The analysis of morphological changes of C6 cells following THC and THC-di-Phe treatments demonstrated similar results with MTT results. The treatment with THC-di-Phe reduced the cell growth and induced condensation of cell cytoplasm and nucleus to a greater extent than THC, indicating its higher antiproliferative potential (Figure 5a; only the photomicrographs resembling the cells treated for 48 h are presented). As shown in Figure 5b, the cell growth increased with the time in the control group, and the cells reached complete confluence 48 h post-treatment. THC-di-Phe significantly inhibited the proliferation ability of C6 cells compared to the control at 24 h and 48 h post-treatment. However, a significant reduction of cell proliferation by THC was only observed 48 h post-treatment. At 48 h post-treatment, the antiproliferative effect of THC-di-Phe was significantly higher than that of THC (Figure 5b). 

### 2.6. The Effects of THC-di-Phe on Cell Migration in C6 Cells

In malignant cancers, cell migration and invasion are key features of metastasis [44]. Hence, the anticancer compounds are expected to reduce cell migration. In line with that, the ability of THC to inhibit wound closure in human osteosarcoma [45] and C6 glioma cells [13] has previously been reported. In this study, the anti-migrative effect of THC-di-Phe was evaluated and compared with THC using the cell scratch assay. As shown in Figure 5c,d, at 24 h, the THC and THC-di-Phe exhibited significantly reduced wound closure: 46.3% and 32.8%, respectively, compared to the control cells (71.4%). At 48 h exposure, the control cells exhibited 100% wound closure, whereas wound closure of THC and THC-di-Phe-treated cells was 81.4% and 65.2%, respectively, indicating the anti-migrative effects of THC and THC-di-Phe. Moreover, THC-di-Phe remarkably reduced wound closure to a greater extent than THC at 24 and 48 h post-treatment. Overall, THC-di-Phe exhibited significantly higher anti-migrative effects on C6 glioma cells compared to THC, indicating increased therapeutic effects of THC by the Phe conjugation.

### 2.7. Involvement of LAT1 in the Activity of THC-di-Phe

LAT1 overexpression is one of the major hallmarks of brain cancer. In glioma, the increased expression of LAT1 is associated with increased requirements for nutrients supplied to the cells. LAT1 uptakes essential amino acids to the cells used for protein synthesis associated with cell differentiation, proliferation, and metastases. Higher expression of LAT1 in invading glioma cells was also found in glioma patients [46]. In addition, high-grade glioma, glioma type III and IV, is associated with a high expression of LAT1 [47]. Therefore, the anticancer agents targeting LAT1, including active compounds conjugated with amino acids, have been used to treat glioma. These compounds act either as substrates or blockers of LAT1, where both pathways can stimulate cellular death. The anticancer agent that acts as a substrate to LAT1 mediates higher transportation of active compounds through LAT1 and modestly lowers the nutrient supply. On the other hand, compounds that act as LAT1 inhibitors bind to LAT1 and block nutrient supply to the cells, leading to cellular death due to nutrient deprivation. In previous studies, co-treatment of compounds with LAT1 inhibitors (BCH) has been used to determine the role of amino acid conjugates on LAT1 activity [48]. Hence, in the present study, THC-di-Phe was co-incubated with BCH, an inhibitor of LAT1, to determine the role of THC-di-Phe on LAT1.

**Figure 6:**
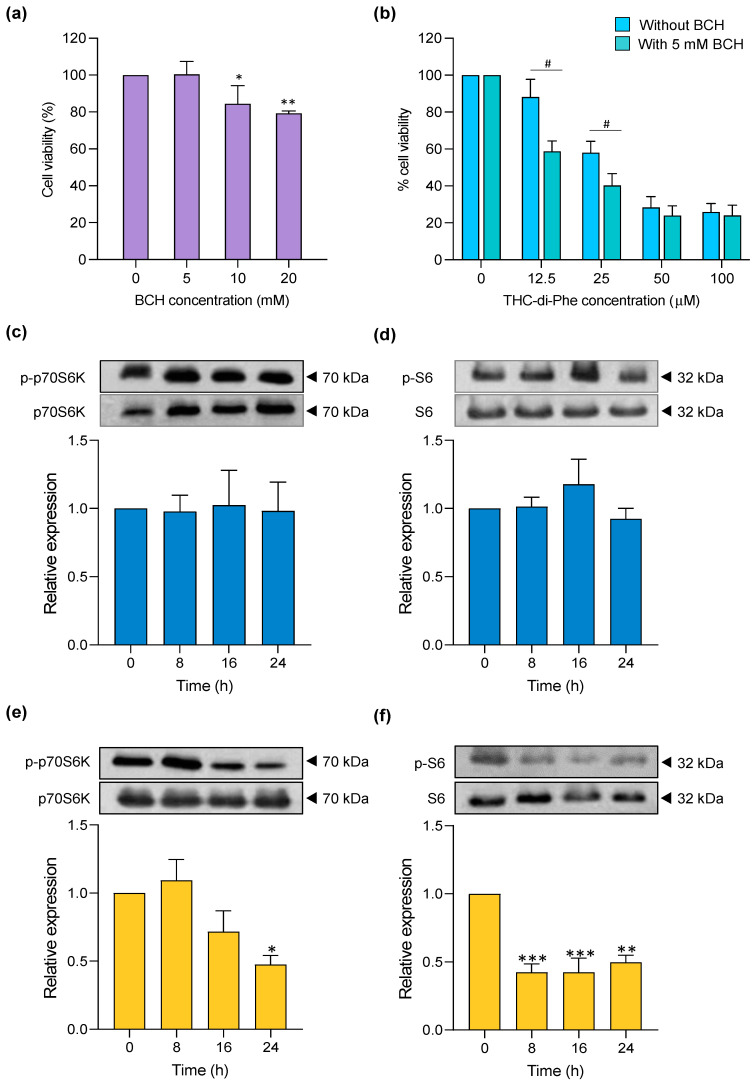
The role of THC-di-Phe on LAT1. (**a**) Cytotoxicity profiles of BCH, (**b**) cytotoxicity of THC-di-Phe with and without BCH incubation. (**c**–**f**) The role of THC-di-Phe in the regulation of the P70S6K/S6 pathway. (**c**,**d**) Phosphorylation profiles of THC and (**e**,**f**) THC-di-Phe at different time frames (0, 8, 16, and 24 h post-treatment). n = 3 independent experiments. Statistical analysis was performed using one-way ANOVA followed by Dunnett’s post hoc test. * *p* < 0.05, ** *p* < 0.01, and *** *p* < 0.001 compared to the control group. The cell viability with or without treatment with BCH was compared using the *t*-test. ^#^ *p* < 0.05 compared to cells treated without BCH.

Initially, the maximum non-toxic concentration of BCH was determined to be 5 mM, which was used in subsequent co-incubation experiments (Figure 6a). In line with our results, BCH at 3 mM concentration was reported to completely inhibit the LAT1 activity in C6 glioma cells with no significant cytotoxicity after 24 h incubation. In contrast, 10 mM of BCH showed significant toxicity [49]. The co-incubation of THC-di-Phe with BCH significantly increased cell death compared to the cells treated only with THC-di-Phe at lower concentrations of THC-di-Phe: 12.5 and 25 μM (Figure 6b). However, THC-di-Phe with or without BCH at higher concentrations (50 and 100 μM) showed comparable cellular death. Overall, the results showed that BCH could not reduce the cytotoxicity of THC-di-Phe but increased the cytotoxic effects of THC-di-Phe, possibly due to the LAT-1 inhibitory effects of THC-di-Phe. Accordingly, it has previously been reported that the phenylalanine conjugated with bulky hydrophobic components could act as LAT1 inhibitors [35]. However, further studies such as competitive [^14^C]-l-leucine (LAT1 substrate) inhibition assays are necessary to confirm the LAT-1 inhibitory role of THC-di-Phe. Moreover, as THC primarily enters cells via passive diffusion due to its lipophilic nature, this may also serve as a potential mechanism for the cellular uptake of THC-conjugates. Further physicochemical characterization of THC-di-Phe is necessary to fully understand its cellular uptake mechanism.

### 2.8. Role of THC-di-Phe in p70S6/S6 Pathway

We examined the expression and phosphorylation of P70S6K and S6, which play an important role in mediating cell death associated with protein metabolism, to examine the ability of THC-di-Phe to induce cellular cell death via the amino acid transporter. LAT1 inhibitors inhibit P70S6K/S6 pathway activity by suppressing the phosphorylation of P70S6K and S6 proteins [50,51]. In this study, the expression of these two proteins and their phosphorylation forms were assessed using immunoblotting. As shown in Figure 6c–f, 10 µM THC did not significantly modulate the phosphorylation of S6 and P70S6K. Conversely, cells treated with 10 µM THC-di-Phe significantly inhibited the phosphorylation of P70S6K and S6 proteins compared to the control group (Supplementary Figures S30 and S31). These results indicate that THC-di-Phe modulates P70S6K and S6 by inhibiting their phosphorylation forms, suggesting its involvement in the modulation of the P70S6K/S6 pathway to induce cell death.

### 2.9. In-Silico Molecular Docking

**Figure 7:**
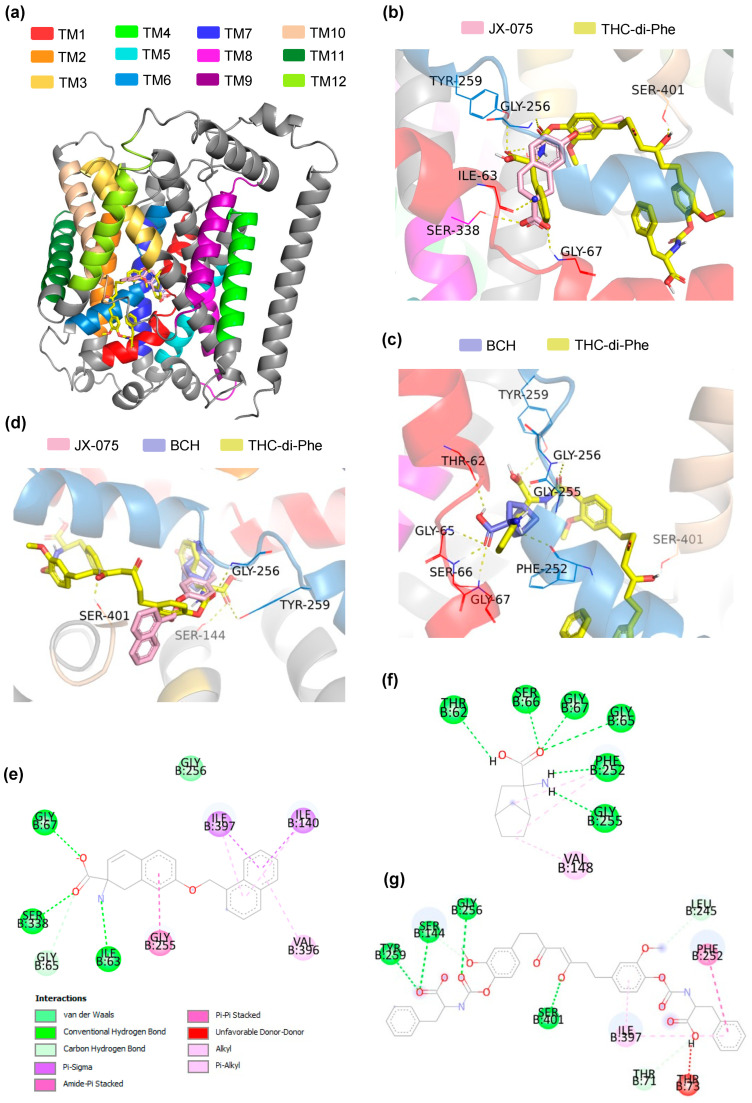
Molecular docking of THC-amino conjugates with LAT1. (**a**) LAT1 transmembrane segments (TMs). (**b**) Overlay binding mode of JX-075 and THC-di-Phe to the outward-occluded conformation of the LAT1 protein. (**c**) Overlay binding mode of JX-075 and THC-di-Phe to the outward-occluded conformation of the LAT1 protein. (**d**) Overview of the overlay binding mode of LAT1 inhibitors, JX-075 and BCH, and THC-di-Phe to the outward-occluded conformation of the LAT1 protein. 2D binding mode showing the interaction of JX-075 (**e**), BCH (**f**), and THC-di-Phe (**g**) with LAT1.

Molecular docking was performed using the AutoDock suite of programs to predict the possible interactions between THC-amino acid conjugates and the LAT1 transmembrane protein, which is a promising target for cancer treatment. The crystal structure of LAT1 bound to a potent inhibitor, JX-075 (PDB code: 7DSK), adopting an outward-occluded conformation, was selected from the RCSB Protein Data Bank, with a resolution of 2.90 Å. It presents a high-resolution crystal structure of the LAT1 inhibitor complex in an outward-occluded conformation, which may be relevant for understanding the mechanism of inhibition and developing new LAT1 inhibitors with improved potency and selectivity. As shown in Figure 7a, LAT1 consists of 12 transmembrane segments (TMs), with the substrate binding site surrounded by TM1, TM3, TM6, TM8, and TM10 [52]. The binding energy and binding modes of THC-di-Phe and known-LAT1 inhibitors (JX-075 and BCH) with the LAT1 protein were summarized in Table 2.

The docking results revealed that JX-075, a recognized LAT1 inhibitor, formed hydrogen bond interactions with ILE63, GLY67 on TM1, and SER338 on TM 8 of the LAT1 receptor (Figure 7b,e), resulting in a notable binding energy of −10.9 kcal/mol. This finding indicates a high affinity for the LAT1 receptor. In contrast, BCH, another established LAT1 inhibitor, also exhibited hydrogen bond interactions with the LAT1 receptor, specifically involving residues THR62, SER66, GLY65, GLY67, PHE252, and PHE255 (Figure 7c,f), albeit with a slightly high binding energy of −6.2 kcal/mol. Intriguingly, THC-di-Phe, the compound under investigation, displayed distinct hydrogen bond interactions with LAT1 residues, namely SER144, GLY256, and TYR259 on TM 6 and SER401 on TM 10 (Figure 7g). In line with that, the hydrogen interaction of LAT1 inhibitors and THC-*di*-Phe was also on TM1 and TM6, which are the substrate binding sites. Therefore, these results indicated that THC-di-Phe could be docked in the same active site of LAT1 compared to JX-075 and BCH (Figure 7d). The calculated binding energy for THC-di-Phe was −9.7 kcal/mol. The observation of hydrogen bond interactions between THC-di-Phe and LAT1, akin to those observed with established LAT1 inhibitors, implies its potential as a LAT1 inhibitor. Moreover, the relatively strong binding energy of THC-di-Phe with the LAT1 receptor suggests a favorable interaction profile. These findings collectively indicate the potential of THC-di-Phe as an inhibitor of LAT1 activity, which may lead to anticancer effects. The binding mode of THC-di-Phe, characterized by hydrogen bond interactions with key LAT1 residues, further supports its ability to interact with the receptor in a manner similar to established LAT1 inhibitors. Therefore, the docking study supported the potential anticancer activity of THC-di-Phe and its efficacy as a LAT1 inhibitor.

## 3. Materials and Methods

### 3.1. Materials

THC was purchased from the Zhonglan industry (Jinan, China). Glycine *tert*-butyl ester hydrochloride (CAS No. 27532-96-3) and bis(4-nitrophenyl) carbonate were obtained from the Tokyo Chemical Industry (TCI, Tokyo, Japan). L-isoleucine *tert*-butyl ester hydrochloride (CAS No. 69320-89-4) and L-leucine *tert*-butyl ester hydrochloride (CAS No. 2748-02-9) were purchased from AK Scientific (AKSci, Union City, CA, USA). L-Phenylalanine *tert*-butyl ester hydrochloride (CAS No. 15100-75-1) was obtained from Combi-Blocks (Combi-Blocks industry, San Diego, CA, USA). Chemicals, solvents, and reagents: ethyl-acetate, dichloromethane (DCM), hexane (RCI, Labscan Bangkok, Thailand), 4-dimethylaminopyridine (DMAP) (Sigma–Aldrich, St. Louis, MO, USA) and sodium sulfate (Merck, Darmstadt, Germany) were purchased. Flash EcoFlex cartridge column (Type, silica; resolution, 40–63 µm; silica weight, 12 g; diameter, 21.7; length, 7 mm) was purchased from Buchi (Bangkok, Thailand). ^1^H and ^13^C NMR spectra were recorded on a Bruker Fourier 400 MHz spectrometer (Bruker, Zurich, Switzerland). High-resolution mass spectra (HRMS) were obtained on a Dionex Ultimate 3000 HPLC (The Thermo Fisher Scientific, Waltham, MA, USA) coupled with a MicroTOF-QII high-resolution mass spectrometer (Bruker, Bremen, Germany).

### 3.2. Synthesis and Structural Elucidation

THC-amino acid carbamate ester conjugates were synthesized in three steps, as shown in Figure 1.

### 3.3. Activated 4-Nitrophenyl Carbamate Intermediate

Amino acid *t*-butyl ester (**a**–**d**) (1.0 eq) and DMAP (2.0 eq) were added dropwise to a solution of bis (4-nitrophenyl) carbonate (1.0 eq) in acetonitrile. The reaction was stirred at 50 °C for 3 h. The reaction mixture was then washed with 0.5 N HCl. The aqueous layer was extracted with dichloromethane (3 × 15 mL), all organic fractions were collected, and anhydrous sodium sulfate was added to remove residual water. Then, the dried dichloromethane layer was concentrated under reduced pressure. The obtained crude product was purified by flash chromatography.

*tert*-Butyl ((4-nitrophenoxy)carbonyl)glycinate (**a**). Purified using flash chromatography: DCM:hexane:acetone (9:1:0.5) as an eluent. 78% yield as a pale-yellow oil. ^1^H NMR (400 MHz, CDCl_3_) δ 8.26 (d, *J* = 9.2 Hz, 2H, H3), 7.34 (d, *J* = 9.2 Hz, 2H, H2), 5.69 (s, 1H, NH), 3.98 (d, *J* = 5.3 Hz, 2H, H1), 1.52 (s, 9H, C(CH_3_)_3_). HRMS calculated for (C_13_H_16_N_2_O_6_Na) [M + Na^+^]: 319.1008; found 319.1020 (Supplementary Figures S13 and S14 and Table S5).

*tert*-Butyl ((4-nitrophenoxy)carbonyl)leucinate (**b**). Purified using flash chromatography: DCM:hexane:acetone (8:2:0.5) as an eluent. 79% yield as a pale-yellow oil. ^1^H NMR (400 MHz, CDCl_3_) δ 8.25 (d, *J* = 9.1 Hz, 2H, H3), 7.34 (d, *J* = 9.1 Hz, 2H, H2), 5.65 (d, *J* = 7.6 Hz, 1H, NH), 4.33 (d, *J* = 6.5 Hz, 1H, H1), 1.88–1.65 (m, 3H, H2 and H4), 1.51 (s, 9H,C(CH_3_)_3_), 1.01 (dd, *J* = 6.5, 2.0 Hz, 6H, H7 and H5). HRMS (ESI+) *m*/*z* calculated for (C_17_H_24_N_2_O_6_Na) 375.1527; found [M + Na^+^]: 375.1525 (Supplementary Figures S15 and S16 and Table S5).

*tert*-Butyl 3-methyl-2-(((4-nitrophenoxy)carbonyl)amino)pentanoate (**c**). Purified using flash chromatography: DCM:hexane (8:2) as an eluent. The residue was loaded on silica and purified using flash chromatography: DCM:hexane:acetone (9:1:0.5). 75% yield as a pale-yellow oil. ^1^H NMR (400 MHz, CDCl_3_) δ 8.26 (d, *J* = 9.1 Hz, 2H, H3), 7.35 (d, *J* = 9.1 Hz, 2H, H2), 5.75 (d, *J* = 8.7, 1H, NH), 4.29 (dd, *J* = 8.7, 4.3 Hz, 1H, H1), 1.97 (m, 1H, H4), 1.52 (s, 9H, C(CH_3_)_3_), 1.28 (s, 2H, H5), 1.03–0.93 (m, 6H, H6 and H7). HRMS (ESI+) *m*/*z* calculated for (C_17_H_24_N_2_O_6_Na) 375.1527; found [M + Na^+^]: 375.1518 (Supplementary Figures S17 and S18 and Table S5).

*tert*-Butyl ((4-nitrophenoxy)carbonyl)phenylalaninate (**d**). Purified using flash chromatography: DCM:acetone (9:1) as an eluent. 82% yield as a pale-yellow oil. ^1^H NMR (400 MHz, acetone-*d*_6_) δ 8.27–8.22 (d, *J*= 9.1 Hz, 2H, H3), 7.33 (d, *J* = 9.1′ Hz, 2H, H2), 7.29–7.24 (m, 5H, H-phenyl), 6.32 (d, *J* = 8.4 Hz, 1H, NH), 4.49–4.40 (m, 1H, H1), 3.12–3.06 (m, 2H, H4), 1.43 (s, 9H, C(CH_3_)_3_). HRMS calculated for (C_20_H_22_N_2_O_6_Na) [M + Na^+^]: 409.1478; found 409.1455 (Supplementary Figures S19 and S20 and Table S5).

### 3.4. Synthesis of Tetrahydrocurcumin-t-Boc-Amino Acid Carbamate Esters

The activated 4-nitrophenyl carbamate intermediate (**a–d**) (2.5 eq) and DMAP (3.0 eq) in dichloromethane were dissolved in 7 mL of dichloromethane. A solution of tetrahydrocucumin (1.0 eq.) in 2 mL of dichloromethane was added gradually and stirred at 50 °C for 24 h. The reaction mixture was then separated with 0.5 N HCl. The aqueous layer was extracted with dichloromethane (3 × 15 mL), all organic layers were combined, and the residual water was removed using anhydrous sodium sulfate. Then, the dichloromethane layer was removed under reduced pressure. The obtained crude product was purified by flash chromatography.

Di-*tert*-butyl 2,2′-(((((3-hydroxy-5-oxohept-3-ene-1,7-diyl)bis(2-methoxy-4,1-phenylene))bis(oxy)) bis(carbonyl))bis(azanediyl))(Z)-diacetate (**1a**). Purified using flash chromatography: DCM:hexane:acetone (7:3:0.5) as an eluent. 67% yield as a colorless oil. ^1^H NMR (400 MHz, CDCl_3_) δ 6.99 (t, *J* = 7.1 Hz, 2H, H9 and H9′), 6.79–6.68 (m, 4H, H6, H6′ and H10 and H10′), 5.88–5.64 (m, 2H, 2 × NH), 5.44 (s, 1H, H1), 3.93 (d, *J* = 5.3 Hz, 4H, H2″), 3.80 (s, 6H, O-CH_3_), 2.95–2.83 (m, 4H, H4 and H4′), 2.83–2.68 (m, 2H, H3), 2.59–2.56 (m, 2H, H3′), 1.49 (s, 18H, C(CH_3_)_3_). HRMS (ESI+) *m*/*z* calculated for (C_35_H_46_N_2_O_12_Na) 709.2943; found [M + Na^+^]: 709.2925 (Supplementary Figures S21 and S22 and Tables S6 and S7).

Di-*tert*-butyl-2,2′-(((((3-hydroxy-5-oxohept-3-ene-1,7-diyl)bis(2-methoxy-4,1-phenylene))bis (oxy))bis(carbonyl))bis(azanediyl))(Z)-bis(4-methylpentanoate) (**1b**). Purified using flash chromatography: DCM:hexane:acetone (2:6:2) as an eluent. 65% yield as an off-white oil. ^1^H NMR (400 MHz, CDCl_3_) δ 7.01 (d, *J* = 7.9 Hz, 2H, H9 and H9′), 6.79–6.73 (m, 4H, H6, H6′ and H10 and H10′), 5.59 (d, *J* = 8.5 Hz, 2H, 2 × NH), 5.46 (s, 1H, H1), 4.34–4.30 (m, 2H, H2″), 3.82 (s, 6H, O-CH_3_), 2.91 (dd, *J* = 8.9, 6.8 Hz, 4H, H4 and H4′), 2.59 (dd, *J* = 8.9, 6.8 Hz, 4H, H3 and H3′), 1.85–1.72 (m, 6H, H3″ and H4″), 1.50 (s, 18H, C(CH_3_)_3_), 1.01–0.98 (m, 12H, H5″ and H6″). HRMS (ESI+) *m*/*z* calculated for (C_43_H_62_N_2_O_12_Na) 821.4195; found [M + Na^+^]: 821.4178 (Supplementary Figures S23 and S24 and Tables S6 and S7).

Di-*tert*-butyl-2,2′-(((((3-hydroxy-5-oxohept-3-ene-1,7-diyl)bis(2-methoxy-4,1-phenylene))bis (oxy))bis(carbonyl))bis(azanediyl))(Z)-bis(3-methylpentanoate) (**1c**). Purified using flash chromatography: DCM:hexane:acetone (2:6:2) as an eluent. 67% yield as an off-white oil. ^1^H NMR (400 MHz, CDCl_3_) δ 7.02 (t, *J* = 6.8 Hz, 2H, H9 and H9′), 6.80–6.73 (m, 4H, H6, H6′, H10 and H10′), 5.67 (d, *J* = 8.6 Hz, 2H, 2 × NH), 5.47 (s, 1H, H1), 4.28 (dd, *J* = 8.6, 4.5 Hz, 2H, H2″), 3.83 (s, 6H, O-CH_3_), 2.91 (dd, *J* = 8.9, 6.8 Hz, 4H, H4 and H4′), 2.60 (dd, *J* = 8.9, 6.8 Hz, 4H, H3 and H3′), 1.97–1.92 (m, 2H, H3″), 1.51 (s, 18H, C(CH_3_)_3_), 1.30–1.25 (m, 4H, H4″), 1.01–0.96 (m, 12H, H5″ and H6″). HRMS (ESI+) *m*/*z* calculated for (C_43_H_62_N_2_O_12_Na) 821.4195; found [M + Na^+^]: 821.4156 (Supplementary Figures S25 and S26 and Tables S6 and S7).

Di-*tert*-butyl-2,2′-(((((3-hydroxy-5-oxohept-3-ene-1,7-diyl)bis(2-methoxy-4,1-phenylene))bis (oxy))bis(carbonyl))bis(azanediyl))(Z)-bis(3-phenylpropanoate) (**1d**). Purified using flash chromatography: DCM:hexane:acetone (5:5:0.5) as an eluent. 66% yield as an off-white oil. ^1^H NMR (400 MHz, acetone-*d*_6_) δ 7.29–7.26 (m, 10H, H-phenyl), 6.89 (d, *J* = 6.5 Hz, 2H, H9 and H9′), 6.81–6.74 (m, 4H, H6, H6′, H10 and H10′), 5.97 (d, *J* = 8.1 Hz, 2H, 2 × NH), 5.68 (s, 1H, H1), 4.53–4.51 (m, 2H, H2″), 3.77 (s, 6H, O-CH_3_), 3.26–3.14 (m, 4H, H3″), 3.01–2.95 (m, 4H, H4 and H4″), 2.66–2.62 (m, 4H, H3, H3′), 1.44 (s, 18H, C(CH_3_)_3_). HRMS calculated for (C_49_H_58_N_2_O_12_Na) [M + Na^+^]: 909.3990; found 909.4010 (Supplementary Figures S27 and S28 and Tables S6 and S7).

### 3.5. Deprotection of t-Boc Group in Tetrahydrocurcumin-t-Boc-Amino Acid Carbamate Esters

Each tetrahydrocurcumin-*t*-Boc-amino acid carbamate ester conjugate (**1a–1d**) (100 mg) was dissolved in dichloromethane 5 mL at 0 °C. To this solution, trifluoroacetic acid (TFA 500 µL) in dichloromethane 1 mL was added slowly under ice-cold conditions. The reaction mixture was stirred at room temperature for 24 h. The solvent and residual TFA were removed under reduced pressure. The residue was triturated with methanol (3 × 15 mL), and the solvent was removed under reduced pressure. The product was purified by flash chromatography.

(Z)-2,2′-(((((3-Hydroxy-5-oxohept-3-ene-1,7-diyl)bis(2-methoxy-4,1 phenylene))bis(oxy))bis (carbonyl))bis(azanediyl))diacetic acid (**2a**). Purified using flash chromatography: DCM:ethyl acetate:formic acid (7:3:0.2) as an eluent. They were further purified via crystallization using ethyl acetate and hexane to obtain an off-white solid with a 75% yield. ^1^H NMR (400 MHz, acetone-*d*_6_) δ 7.03–6.86 (m, 4H, H6, H6′, H10 and H10′), 6.83–6.67 (m, 2H, H9 and H9′), 5.54 (s, 1H, H1), 3.91 (s, 4H, H2″), 3.78 (s, 6H, O-CH_3_), 2.94–2.69 (m, 6H, H3 and H4, H4′), 2.60 (d, *J* = 7.5 Hz, 2H, H3′). ^13^C NMR (101 MHz, methanol-d_4_) δ 204.90, 192.95, 171.98, 156.00, 151.65, 151.65, 139.61, 139.61, 138.31, 135.31, 122.60, 122.60, 119.98, 119.98, 112.59, 112.59, 100.15, 55.05, 44.51, 42.00, 40.46, 33.89, 33.09. HRMS (ESI+) *m*/*z* calculated for (C_27_H_30_N_2_O_12_Na) 597.1691; found [M + Na^+^]: 597.1678 (Supplementary Figures S1–S3 and Tables S1 and S3).

(Z)-2,2′-(((((3-Hydroxy-5-oxohept-3-ene-1,7-diyl)bis(2-methoxy-4,1-phenylene))bis(oxy))bis (carbonyl))bis(azanediyl))bis(4-methylpentanoic acid) (**2b**). Purified using flash chromatography: DCM:ethyl acetate:formic acid (8:2:0.2) as an eluent. They were further purified via crystallization using ethyl acetate and hexane to obtain an off-white solid with a 90% yield. ^1^H NMR (400 MHz, acetone-*d*_6_) δ 6.96–6.91 (m, 4H, H6, H6′, H10 and H10′), 6.80–6.70 (m, 2H, H9 and H9′), 5.67 (s, 1H, H1), 4.34–4.25 (m, 2H, H2″), 3.77 (s, 6H, O-CH_3_), 2.91–2.82 (m, 4H, H4 and H4′), 2.76–2.45 (m, 4H, H3 and H3′), 1.73–1.60 (m, 6H, H3″ and H4″), 1.07–0.95 (m, 12H, H5″ and H6″). ^13^C NMR (101 MHz, acetone-d_6_) δ 203.57, 193.32, 174.29, 158.06, 154.35, 154.35, 139.48, 139.48, 139.19, 139.19, 122.85, 122.85, 120.00, 120.00, 112.85, 112.85, 99.49, 55.29, 52.52, 44.62, 40.78, 39.53, 31.75, 31.75, 24.56, 21.04, 21.04. HRMS (ESI+) *m*/*z* calculated for (C_35_H_46_N_2_O_12_Na) 709.2943; found [M + Na^+^]: 709.2920 (Supplementary Figures S4–S6 and Tables S1 and S3).

(Z)-2,2′-(((((3-Hydroxy-5-oxohept-3-ene-1,7-diyl)bis(2-methoxy-4,1-phenylene))bis(oxy))bis (carbonyl))bis(azanediyl))bis(3-methylpentanoic acid) (**2c**). Purified using flash chromatography: DCM:ethyl acetate:formic acid (8:2:0.2) as an eluent. They were further purified via crystallization using ethyl acetate and hexane to obtain an off-white solid with an 85% yield. ^1^H NMR (400 MHz, acetone-*d*_6_) δ 6.96–6.92 (m, 4H, H6, H6′, H10 and H10′), 6.78–6.76 (m, 2H, H9 and H9′), 5.68 (s, 1H, H1), 4.25–4.21 (m, 2H, H2″), 3.77 (s, 6H, O-CH_3_), 2.92–2.82 (m, 6H, H3 and H4, H4′), 2.65–2.61 (m, 2H, H3′), 2.00–1.94 (m, 2H, H3″), 1.40–1.29 (m, 4H, H4″), 0.97–0.86 (m, 12H, H5″ and H6″). ^13^C NMR (101 MHz, acetone-d_6_) δ 204.52, 194.26, 173.32, 155.35, 152.74, 152.74, 140.51, 140.51, 140.17, 140.17, 123.79, 123.79, 120.95, 120.95, 113.78, 113.78, 100.42, 59.60, 56.24, 45.55, 40.45, 38.30, 31.94, 31.94, 25.73, 16.04, 11.89. HRMS (ESI+) *m*/*z* calculated for (C_35_H_46_N_2_O_12_Na) 709.2943; found [M + Na^+^]: 709.2926 (Supplementary Figures S7–S9 and Tables S2 and S3).

(Z)-2,2′-(((((3-Hydroxy-5-oxohept-3-ene-1,7-diyl)bis(2-methoxy-4,1-phenylene))bis(oxy))bis (carbonyl))bis(azanediyl))bis(3-phenylpropanoic acid) (**2d**). Purified using flash chromatography: DCM: ethyl acetate: formic acid (9:1:0.2) as an eluent. They were further purified via crystallization using ethyl acetate and hexane to obtain the off-white solid with a 77% yield. ^1^H NMR (400 MHz, acetone-*d*_6_) δ 7.39–7.32 (m, 10H, H-phenyl), 6.95 (d, *J* = 2.1 Hz, 2H, H6 and H6′), 6.81–6.68 (m, 4H, H9, H9′, H10 and H10′), 5.63 (s, 1H, H1), 4.54–4.51 (m, 2H, H2″), 3.75 (s, 6H, O-CH_3_), 3.44–3.17 (m, 4H, H3″), 3.15–2.97 (m, 4H, H4 and H4′), 2.69–2.57 (m, 4H, H3 and H3′). ^13^C NMR (101 MHz, methanol-d_4_) δ 204.74, 192.85, 173.97, 155.13, 151.63, 151.63, 139.48, 139.48, 138.31, 137.28, 137.28, 129.25, 126.35, 126.35, 122.55, 122.55, 112.59, 112.59, 106.72, 55.03, 44.45, 39.37, 38.81, 37.32, 37.32, 30.98. HRMS (ESI+) *m*/*z* calculated for (C_41_H_42_N_2_O_12_Na) 777.2629; found [M + Na^+^]: 777.2598 (Supplementary Figures S10–S12 and Tables S2 and S3).

### 3.6. Cell Culture

C6 glioma cells and BJ fibroblast cells (ATCC, Manassas, VA, USA) were used as models for brain cancer and normal cells, respectively. The cells were cultured in DMEM media (Sigma–Aldrich, St. Louis, MO, USA) supplemented with 10% fetal bovine serum (Merck Millipore, Burlington, MA, USA) and 1% penicillin-streptomycin (Caisson Labs, Smithfield, UT, USA). The cells were maintained in an incubator with 5% CO_2_ at 37 °C and 95% humidity.

### 3.7. Cytotoxicity Profiling of THC-Amino Acid Conjugates

Cell viability was assessed using the 3-(4,5-dimethylthiazol-2-yl)-2,5-diphenyltetrazolium bromide (MTT) assay (Sigma–Aldrich, St. Louis, MO, USA). Cells were seeded in 96-well plates at a density of 2 × 10^4^ cells/well and incubated for 24 h. The cells were then treated with various concentrations of THC and equimolar concentrations of THC-amino acid conjugates (0, 31.25, 62.5, 125, 250, and 500 μM) for 24 and 48 h in cancer cells, and for 24 h in normal cells. After treatment, the media were removed and replaced with MTT solution (0.5 mg/mL), followed by incubation for 3 h. The resulting formazan crystals were dissolved in DMSO (Sigma–Aldrich, St. Louis, MO, USA). Absorbance was measured using a microplate reader at a wavelength of 570 nm (CLARIOstar^®^, BMG Labtech, Ortenberg, Germany).

### 3.8. Assessment of Apoptosis and Necrosis

Apoptosis and necrosis were assessed using the Hoechst 33342/PI double staining assay (Sigma–Aldrich, St. Louis, MO, USA). After treatment, the cells were viewed using a fluorescence microscope (Olympus IX51 inverted microscope, Tokyo, Japan). Hoechst 33342 dye stains cells with blue color, either dead or alive. Apoptotic cells are indicated by condensed and fragmented chromatin. Propidium iodide (PI) stains cells in red, which can only stain damaged cells and is used as an indicator of cellular necrosis [53].

### 3.9. Flow Cytometry

Annexin V/PI staining by flow cytometry was performed to indicate the stage of cell fate, including necrosis, early and late apoptosis, and viable cells. Briefly, the cells were treated with the THC and THC-di-Phe at 20 µM. After 24 h, the cells were harvested and further analyzed by flow cytometry. Annexin V/PI staining by flow cytometry was performed according to the manufacturer’s instructions (BioLegend, San Diego, CA, USA).

### 3.10. The Effects of THC-di-Phe on Cancer-like Behaviors

#### 3.10.1. Cell Proliferation Assay

The effects of THC-di-Phe on cell cancer proliferation were assessed using a cell proliferation assay. Briefly, C6 cells were seeded in a 96-well plate at a concentration of 5000 cells/well and then incubated overnight. Cells were then incubated with 10 µM THC and THC-di-Phe for 24 and 48 h. The morphology of the cells was observed under a light microscope. Then, the cells were incubated with MTT solution (0.5 mg/mL), and the formed formazan crystals were dissolved in DMSO. The absorbance was measured using a microplate reader (CLARIOstar^®^, BMG Labtech, Ortenberg, Germany) at the wavelength of 570 nm.

#### 3.10.2. Scratch Assay

Properties of cell migration were assayed using a cell scratch assay. Briefly, the cells were seeded in 24-well plates at a concentration of 100,000 cells/well, followed by treatment of the cells using 10 µM THC and 10 µM THC-di-Phe. The cell layers were then scrapped with a 200 µL pipette tip to form a wound. The ability of the cells to close the wound was observed under an inverted microscope (Nikon TS2, Tokyo, Japan) at 0, 24, and 48 h post-treatment. Wound closure was further analyzed using ImageJ software (version 1.53e, Wayne Rasband and contributors, National Institutes of Health, USA).

### 3.11. Investigation of the Role of THC-Amino Acid Conjugates on LAT1

To investigate the role of THC-conjugated amino acid conjugates on LAT1, THC-amino acid conjugates were co-incubated with an inhibitor of LAT1 (BCH). The cells were seeded overnight at 2 × 10^4^ cells/well in 96-well plates. Initially, the cells were treated with different concentrations of BCH (5, 10, and 20 mM) for 24 h, followed by the MTT assay to identify the maximum non-toxic concentration of BCH. Then, the cells were treated with THC-di-Phe at 12.5, 25, and 50 μM concentrations with or without 5 mM BCH for 24 h. The cell viability was measured by MTT assay.

### 3.12. Western Blot Analysis

C6 cells were seeded in a 6-well plate with a density of 1 × 10^6^ cells for 24 h. The cells were treated with THC and THC-di-Phe for 8, 16, and 24 h. The cells were washed in ice-cold phosphate-buffered saline (PBS) and lysed with ice-cold lysis buffer containing protease and phosphatase inhibitors. The cells in the lysis buffer were centrifuged at 14,000 rpm at 4 °C for 10 min. The protein concentration in the supernatant was quantified using Pierce™ BCA protein assay. After the quantification, equal amounts of protein were resolved by SDS–PAGE and transferred into a nitrocellulose membrane. The membranes were then blocked with 5% dry milk and probed with primary antibody including p70S6K, p-p70S6K, S6, p-S6 (Cell Signaling Technology (Beverly, MA, USA), followed by adding the species-specific horseradish peroxide conjugated secondary antibody (Cell Signaling Technology (Beverly, MA, USA). The membrane was incubated with a chemiluminescent detection kit solution, followed by X-ray film imaging to visualize protein levels. The level of phosphorylation of each protein was normalized to its normal form.

### 3.13. In Silico Molecular Docking Studies

A molecular docking study was performed using AutoDock Vina version 1.2.0. software to characterize the binding mode of THC-di-Phe to LAT1. The lowest binding energy was selected and visualized using UCSF Chimera (The UCSF Resource for Biocomputing, Visualization, and Informatics., University of California, San Francisco). The outward-occluded conformation of LAT1 in complex with a potent LAT1 inhibitor, JX-075 (PDB code: 7DSK), at 2.90 Å resolution was chosen from the RCSB Protein Data Bank. The ligand and water molecules were removed, followed by adding all polar hydrogen atoms. Gasteiger charges were assigned to the proteins using AutoDockTools-1.5.6 (ADT). The cubic box of 80 × 80 × 80 with a grid spacing of 0.375 Å, based on the center (X, Y, Z) of 144.325, 136.877, and 135.073, respectively. For the preparation of the ligands, the 2D and 3D structures of THC-di-Phe, BCH, and JX-075 (LAT1 inhibitor) were prepared and optimized in ACDlab. All ligands were then saved in PDBQT format in ADT software version 1.5.6. In the semiflexible docking protocol, the protein molecule was kept rigid while the ligand was flexible by the rotational parameter. Docking results were visualized using Biovia Discovery Studio Visualizer (Dassault Systèmes BIOVIA Corp., San Diego, CA, USA).

### 3.14. Statistical Analysis

Data are expressed as mean ± SD. The comparison between groups was performed using either ANOVA or the student *t*-test. *p* < 0.05 was considered statistically significant. Statistical analysis was performed using GraphPad Prism version 10.3.0 for Mac (GraphPad Software, Boston, MA, USA).

## 4. Conclusions

In this study, THC, which has limited water solubility, is expected to exhibit improved solubility through conjugation with amino acids via carbamate bonds, introducing polar carboxyl groups. To further enhance solubility, these conjugates can be formulated as salts using bases such as sodium, potassium, arginine, or lysine. Additionally, encapsulation techniques, such as polymeric nanoparticles, liposomes, micelles, hydrogels, or cyclodextrin complexes, are essential to improve stability, bioavailability, and controlled release. Combining salt formation with encapsulation ensures effective delivery of THC conjugates for oral, topical, or injectable applications, addressing solubility challenges and enhancing therapeutic potential.

In conclusion, our results demonstrate increased antitumor effects of THC-di-Phe compared to THC against glioma. THC-di-Phe significantly enhanced the cytotoxic, antiproliferative, and anti-migrative effects of THC in C6 glioma cells. THC-di-Phe with LAT1 inhibitor BCH showed more cytotoxic effects than without BCH in C6 glioma cells. In addition, THC-di-Phe significantly inhibited P70S6K and S6 phosphorylation and showed similar docking alignment with JX-075 and BCH to the active site of LAT1, suggesting its LAT1 inhibitory role. Overall, our results provide the initial experimental insights into the potential use of THC-di-Phe as a more effective treatment against glioma. However, future in vitro and in vivo studies are warranted to evaluate the physicochemical properties, pharmacokinetics, and LAT1 inhibitory activity of THC-di-Phe before translating it to clinical trials as a novel therapeutic lead for glioma.

## Figures and Tables

**Table 1:** The percentage yield of synthesized THC-amino acid conjugates.

Compound	Structure	Formula	MW	% Yield
THC-di-Gly	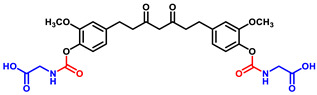	C_27_H_30_N_2_O_12_	574.5390	75.0
THC-di-Leu	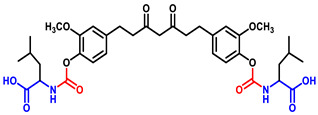	C_35_H_46_N_2_O_12_	686.7550	90.0
THC-di-Ile	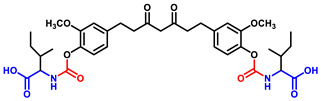	C_35_H_46_N_2_O_12_	686.7550	85.0
THC-di-Phe	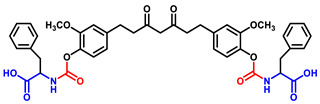	C_41_H_42_N_2_O_12_	754.7890	77.0

**Table 2:** Summary of binding modes and energies of LAT1 complexed with LAT1 inhibitors, JX-075 and BCH, and THC-di-Phe.

Compound	Interaction	Residue	ΔG (kcal/mol)
JX-075	H-bond Carbon H-bond Van der Waals Hydrophobic	ILE63, GLY67, SER338 GLY65 ILE140, GLY255, GLY256, ILE397 ILE140, VAL396, ILE397	−10.9
BCH 2-Aminobicyclo-(2,2,1)-heptane-2-carboxylic acid	H-bond Hydrophobic	THR62, SER66, GLY65, GLY67, PHE252, PHE255 VAL148, PHE252	−6.2
THC-di-Phe	H-bond Carbon H-bond Van der Waals Hydrophobic	SER144, GLY256, TYR259, SER401 THR71, LEU245 PHE252 ILE397	−9.7

## Data Availability

All data relevant to the publication are included.

## Supplementary Materials

The supporting information can be downloaded at: https://www.mdpi.com/article/10.3390/ijms252011266/s1.
