# Supplementary material for: Synthesis, Cytotoxicity, and Mechanistic Evaluation of Tetrahydrocurcumin-Amino Acid Conjugates as LAT1-Targeting Anticancer Agents in C6 Glioma Cells
Source: Int J Mol Sci. 2024 Oct 19;25(20):11266. doi: 10.3390/ijms252011266 (PMC11509005; doi:10.3390/ijms252011266)
Supplement: Supplementary file 1 [file ijms-25-11266-s001.zip › ijms-3230143-supplementary_proofread.pdf]

International Journal of
Molecular Sciences

MDPI

Article

Synthesis, Cytotoxicity, and Mechanistic Evaluation of Tetrahydrocurcumin-Amino Acid Conjugates as LAT1-Targeting Anticancer Agents in C6 Glioma Cells

Polsak Teerawonganan[1,2], Hasriadi[1,3], Peththa Wadu Dasuni Wasana[4], Pornpoom Angsuwattana[5], Apichart Suksamrarn[6], Nonthaneth Nalinratana[1,3], Opa Vajragupta[1,7], Pasarapa Towiwat[1,3], Worathat Thitikornpong[1,5] & Pornchai Rojsitthisak[1,5] *

Supplementary Information

List of Tables

Table S1 1H-NMR (400MHz, compounds 2a-2b in acetone-d6 (δ in ppm, *J* in Hz) 3

Table S2 ^{1}H-NMR (400MHz, compounds 2c-2d in acetone-d6 (δ in ppm, *J* in Hz) 7

Table S3 Molecular weight of compounds 2a-2d detected by HRMS. 11

Table S4 ^{1}H-NMR (400MHz, compounds a-d in $CDCl_3$ and acetone-d6 (δ in ppm, *J* in Hz) .**Error! Bookmark not defined.**

Table S5 Molecular weight of compounds a-d detected by HRMS. .... **Error! Bookmark not defined.**

Table S6 ^{1}H-NMR (400MHz, compound 1a-1d in $CDCl_3$ and acetone-d6 (δ in ppm, J in Hz)..................**Error! Bookmark not defined.**

Table S7 Molecular weight of compounds 1a-1d detected by HRMS. 23

List of Figures

Figure S1 ^{1}H NMR spectrum of tetrahydrocurcumin-di-glycine (2a) 4
Figure S2 ^{13}C NMR spectrum of tetrahydrocurcumin-di-glycine (2a)....................4
Figure S3 Mass spectrum of tetrahydrocurcumin-di-glycine (2a) 5
Figure S4 ^{1}H NMR spectrum of tetrahydrocurcumin-di-leucine (2b) 5
Figure S5 ^{13}C NMR spectrum of tetrahydrocurcumin-di-leucine (2b)....................6
Figure S6 Mass spectrum of tetrahydrocurcumin-di-leucine (2b) 6
Figure S7 ^{1}H-NMR spectrum of tetrahydrocurcumin-di-isoleucine (2c) 8
Figure S8 ^{13}C NMR spectrum of tetrahydrocurcumin-di-isoleucine (2c)....................8
Figure S9 Mass spectrum of tetrahydrocurcumin-di-isoleucine (2c)....................9
Figure S10 ^{1}H NMR spectrum of tetrahydrocurcumin-di-phenylalanine (2d) 9
Figure S11 ^{13}C NMR spectrum of tetrahydrocurcumin-di-phenylalanine (2d)....................10
Figure S12 Mass spectrum of tetrahydrocurcumin-di-phenylalanine (2d)....................10
Figure S13 ^{1}H NMR spectrum of glycine activation (a)....................13
Figure S14 Mass spectrum of glycine activation (a)....................13
Figure S15 ^{1}H NMR spectrum of leucine activation (b)....................14
Figure S16 Mass spectrum of leucine activation (b)....................14
Figure S17 ^{1}H NMR spectrum of isoleucine activation (c) 15
Figure S18 Mass spectrum of isoleucine activation (c) 15
Figure S19 ^{1}H NMR spectrum of phenylalanine activation (d)....................16
Figure S20 Mass spectrum of phenylalanine activation (d)....................16
Figure S21 ^{1}H NMR spectrum of tetrahydrocurcumin-di-glycineBOC (1a)....................19
Figure S22 Mass spectrum of tetrahydrocurcumin-di-glycineBOC (1a)....................19
Figure S23 ^{1}H NMR spectrum of tetrahydrocurcumin-di-leucineBOC (1b)....................20
Figure S24 Mass spectrum of tetrahydrocurcumin-di-leucineBOC (1b)....................20
Figure S25 ^{1}H NMR spectrum of tetrahydrocurcumin-di-isoleucineBOC (1c) 21
Figure S26 Mass spectrum of tetrahydrocurcumin-di-isoleucine BOC (1c) 21
Figure S27 ^{1}H NMR spectrum of tetrahydrocurcumin-di-phenylalanineBOC (1d)....................22
Figure S28 Mass spectrum of tetrahydrocurcumin-di-phenylalanineBOC (1d)....................22
Figure S29 Cytotoxicity profiles of THC-amino acid conjugates in C6 glioma cells. 23
Figure S30 RAW western blot images of THC on the proteins of the P70S6K/S6 pathway at different time frames (0, 8, 16, and 24 h post-treatment). 24
Figure S31 RAW western blot images of THC-di-Phe on the proteins of the P70S6K/S6 pathway at different time frames (0, 8, 16, and 24 h post-treatment). 25

Table S1 1H-NMR (400MHz, compounds 2a-2b in acetone-d6 (δ in ppm, *J* in Hz)

Position	2a		2b	
	**^{1}H signal δ$_H$ (mult., *J*$_{Hz}$)**	**^{13}C signal δ$_C$ (Type)**	**^{1}H signal δ$_H$ (mult., *J*$_{Hz}$)**	**^{13}C signal δ$_C$ (Type)**
O-CH$_3$	3.78 s	55.05 (CH$_3$)	3.77 s	55.29 (CH$_3$)
1	5.54 s	100.15 (CH)	5.65 s	99.49 (CH)
2	-	204.90 (C)	-	203.57 (C)
3	2.69-2.94 m	42.00 (CH$_2$)	2.45-2.76 m	40.78 (CH$_2$)
4	2.69-2.94 m	33.09 (CH$_2$)	2.82-2.91 m	31.75 (CH$_2$)
5	-	139.61 (C)	-	139.48 (C)
6	6.86-7.03 m	112.59 (CH)	6.91-6.96 m	112.85 (CH)
7	-	151.65 (C)	-	154.35 (C)
8	-	135.31 (C)	-	139.19 (C)
9	6.67-6.85 m	119.98 (CH)	6.70-6.80 m	120.00 (CH)
10	6.86-7.03 m	122.60 (CH)	6.91-6.96 m	122.85 (CH)
2′	-	192.95 (C)	-	193.32 (C)
3′	2.60 d (7.5)	40.46 (CH$_2$)	2.45-2.76 m	39.53 (CH$_2$)
4′	2.69-2.94 m	33.89 (CH$_2$)	2.82-2.91 m	31.75 (CH$_2$)
5′	-	139.61 (C)	-	139.48 (C)
6′	2.86-7.03 m	112.59 (CH)	6.91-6.96 m	112.85 (CH)
7′	-	151.65 (C)	-	154.35 (C)
8′	-	138.31 (C)	-	139.19 (C)
9′	6.67-6.85 m	119.98 (CH)	6.70-6.80 m	120.00 (CH)
10′	6.86-7.03 m	122.60 (CH)	-	122.85 (CH)
1″	-	156.00 (C)	-	158.06 (C)
2″	3.41 s	44.51 (CH$_2$)	4.25-4.34 m	52.52 (CH)
3″	-	171.98 (C)	1.60-1.73 m	44.62 (CH$_2$)
4″	-	-	1.60-1.73 m	24.56 (CH)
5″	-	-	0.95-1.07 m	21.04 (CH$_3$)
6″	-	-	0.95-1.07 m	21.04 (CH$_3$)
7″	-	-	-	174.29 (C)

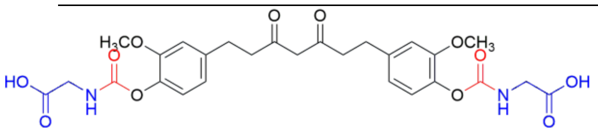

2a

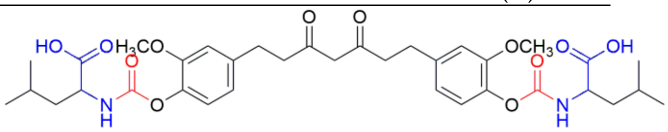

2b

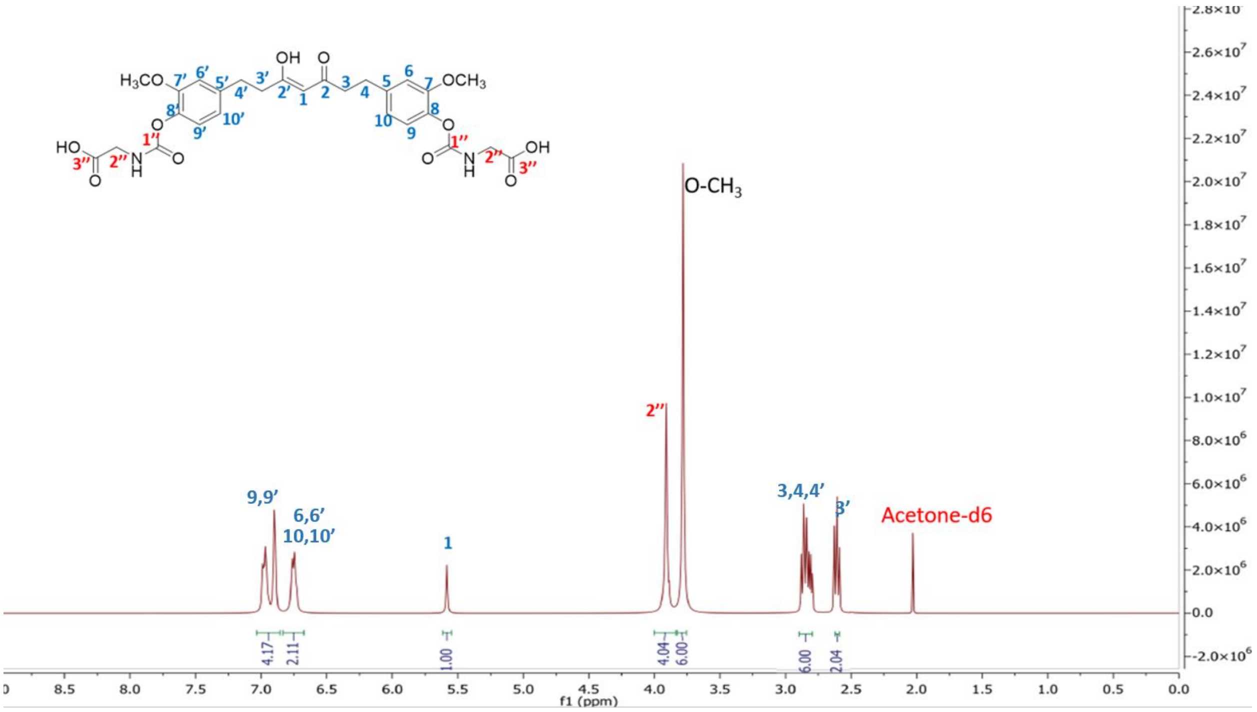

Figure S1 ^{1}H NMR spectrum of tetrahydrocurcumin-di-glycine (2a)

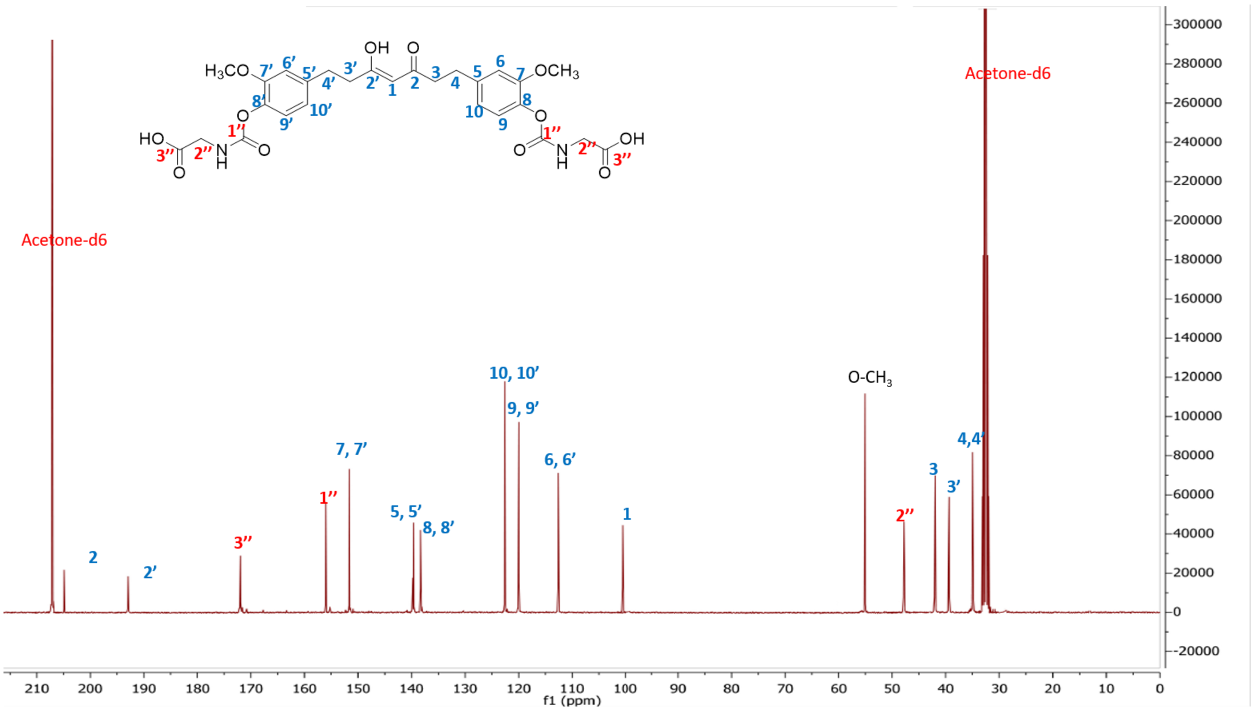

Figure S2 ^{13}C NMR spectrum of tetrahydrocurcumin-di-glycine (2a)

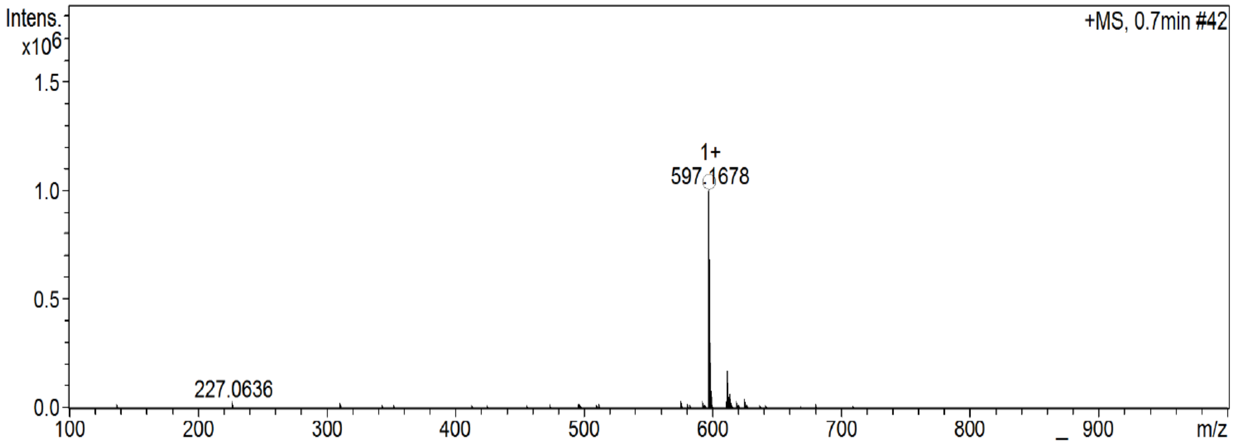

Figure S3 Mass spectrum of tetrahydrocurcumin-di-glycine (2a)

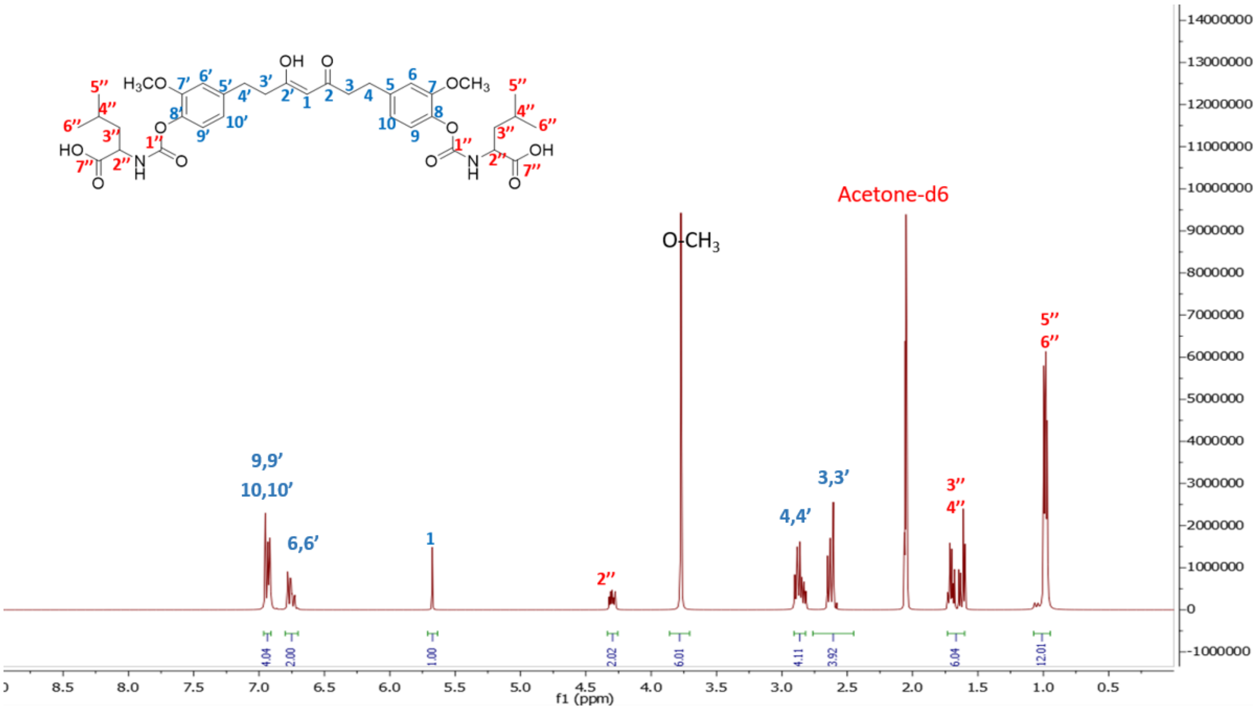

Figure S4 ^{1}H NMR spectrum of tetrahydrocurcumin-di-leucine (2b)

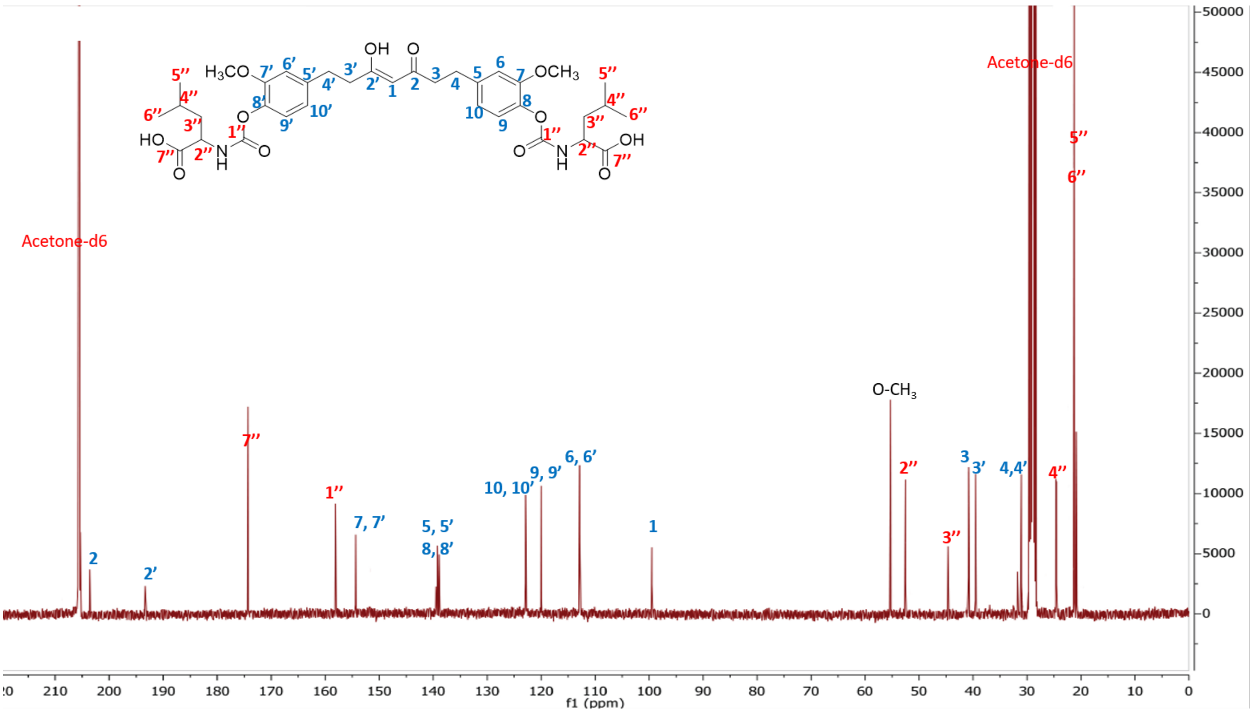

Figure S5 ^{13}C NMR spectrum of tetrahydrocurcumin-di-leucine (2b)

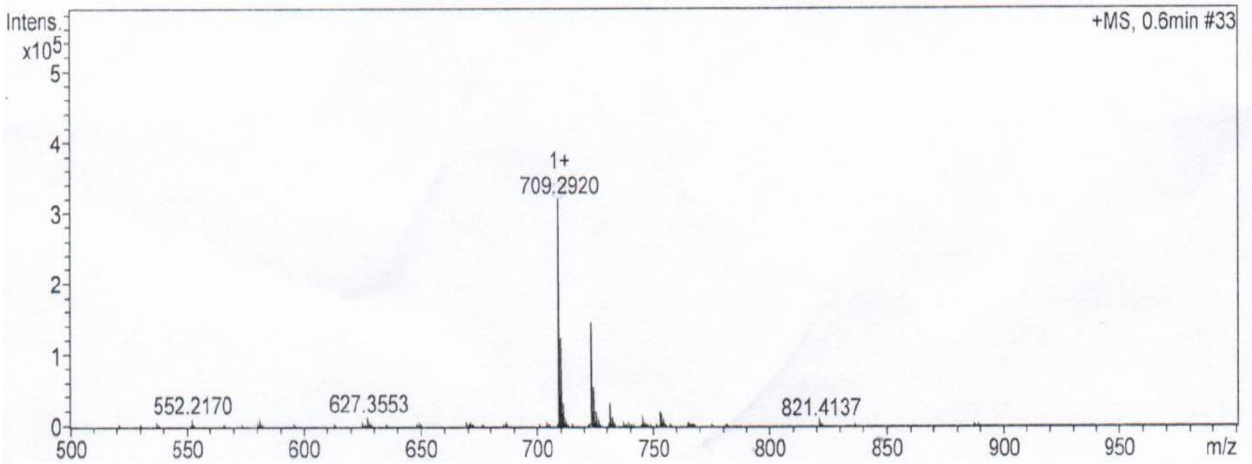

Figure S6.

Figure S6 Mass spectrum of tetrahydrocurcumin-di-leucine (2b)

Table S2 ^{1}H-NMR (400MHz, compounds 2c-2d in acetone-d6 (δ in ppm, *J* in Hz)

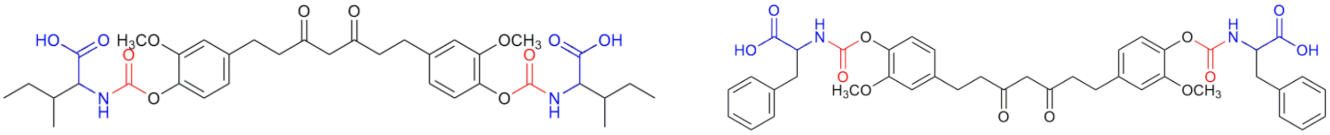

2c 2d

Position	2c		2d	
	**^{1}H signal δ$_H$ (mult., J_{Hz})**	**^{13}C signal δ$_C$ (Type)**	**^{1}H signal δ$_H$ (mult., J_{Hz})**	**^{13}C signal δ$_C$ (Type)**
O-CH_3	δ 3.77 s	56.24 (CH_3)	δ 3.75 s	55.03 (CH_3)
1	δ 5.68 s	100.42 (CH)	δ 5.63 s	106.72 (CH)
2	-	204.52 (C)	-	204.74 (C)
3	δ 2.82-2.92 m	45.55 (CH_2)	δ 2.57-2.69 m	39.37 (CH_2)
4	δ 2.82.2.92 m	31.94 (CH_2)	δ 2.97-3.15 m	37.32 (CH_2)
5	-	140.51 (C)	-	137.28 (C)
6	δ 6.92-6.96 m	113.78 (CH)	δ 6.95 d (2.1)	112.59 (CH)
7	-	152.74 (C)	-	151.63 (C)
8	-	140.17 (C)	-	139.48 (C)
9	δ 6.76-6.78 m	120.95 (CH)	δ 6.68-6.81 m	126.35 (CH)
10	δ 6.92-6.96 m	123.79 (CH)	δ 6.68-6.81 m	122.55 (CH)
2′	-	194.26 (C)	-	192.85 (C)
3′	δ 2.61-2.73 m	40.45 (CH_2)	δ 2.57-2.69 m	38.81 (CH_2)
4′	δ 2.82-2.92 m	31.94 (CH_2)	δ 2.97.3.15 m	37.32 (CH_2)
5′	-	140.51 (C)	-	137.28 (C)
6′	δ 6.92-6.96 m	113.78 (CH)	δ 6.95 d (2.1)	112.59 (CH)
7′	-	152.74 (C)	-	151.63 (C)
8′	-	140.17 (C)	-	139.48 (C)
9′	δ 6.76-6.78 m	120.95 (CH)	δ 6.68-6.81 m	126.35 (CH)
10′	δ 6.92-6.96 m	123.79 (CH)	δ 6.68-6.81 m	122.55 (CH)
1″	-	155.35 (C)	-	155.13 (C)
2″	δ 4.21-4.25 m	59.60 (CH)	δ 4.51-5.54 m	44.45 (CH)
3″	δ 1.94-2.00 m	38.30 (CH)	δ 3.17-3.44 m	30.98 (CH_2)
4″	δ 1.29-1.40 m	25.73 (CH_2)	-	138.31 (C)
5″	δ 0.86-0.97 m	11.89 (CH_3)	-	-
6″	δ 0.86-0.97 m	16.04 (CH_3)	-	-
7″	-	173.32 (C)	-	-
10″		**-**	-	173.97 (C)
Phenyl	-	-	δ 7.32-7.39 m	129.25 (CH)

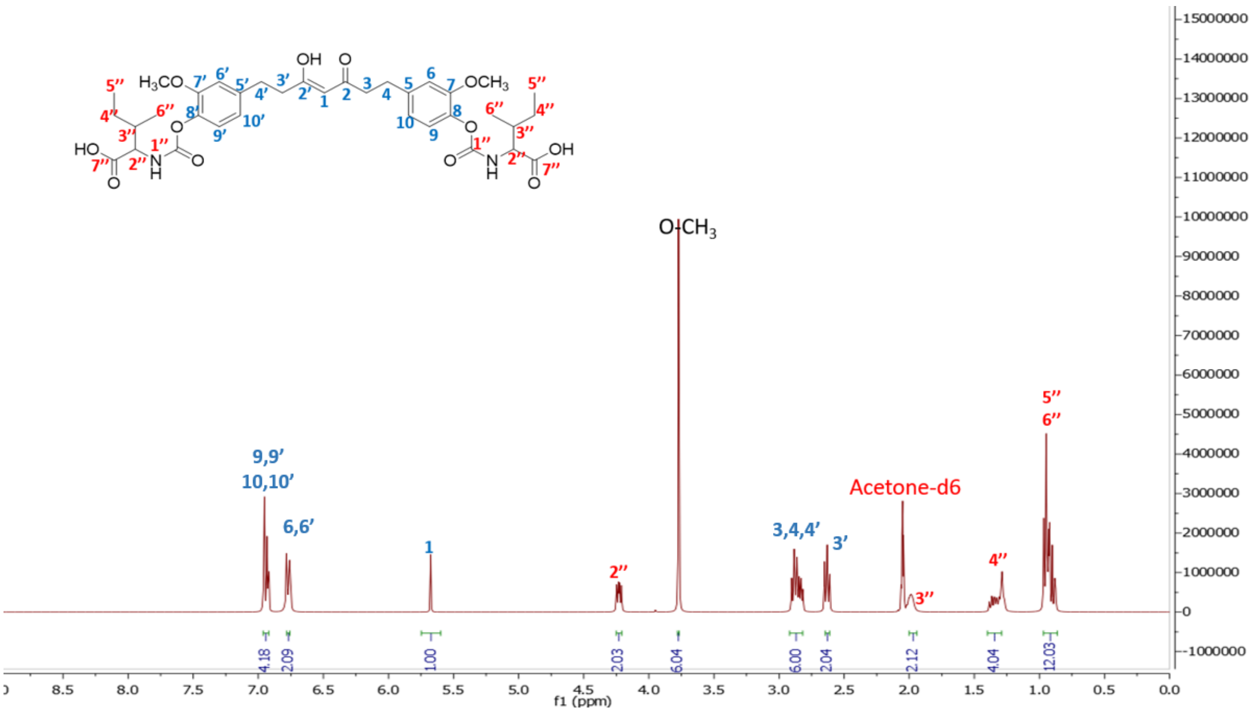

Figure S7 ^{1}H-NMR spectrum of tetrahydrocurcumin-di-isoleucine (2c)

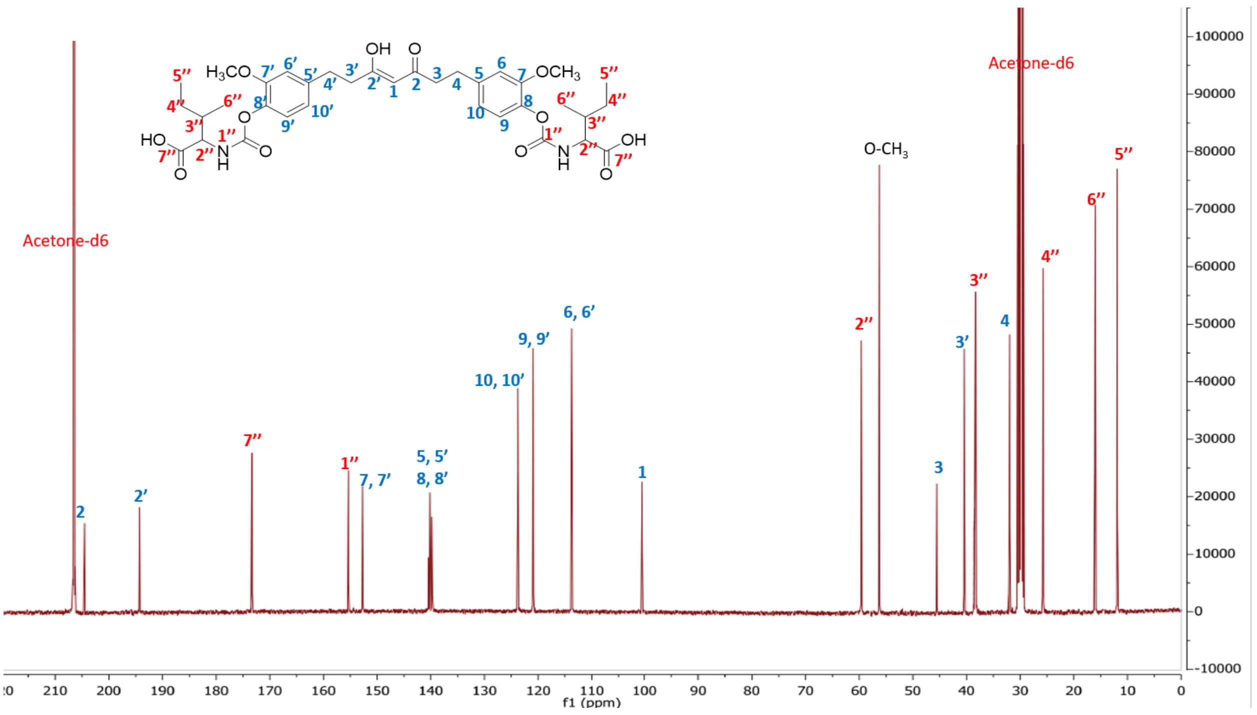

Figure S8 ^{13}C NMR spectrum of tetrahydrocurcumin-di-isoleucine (2c)

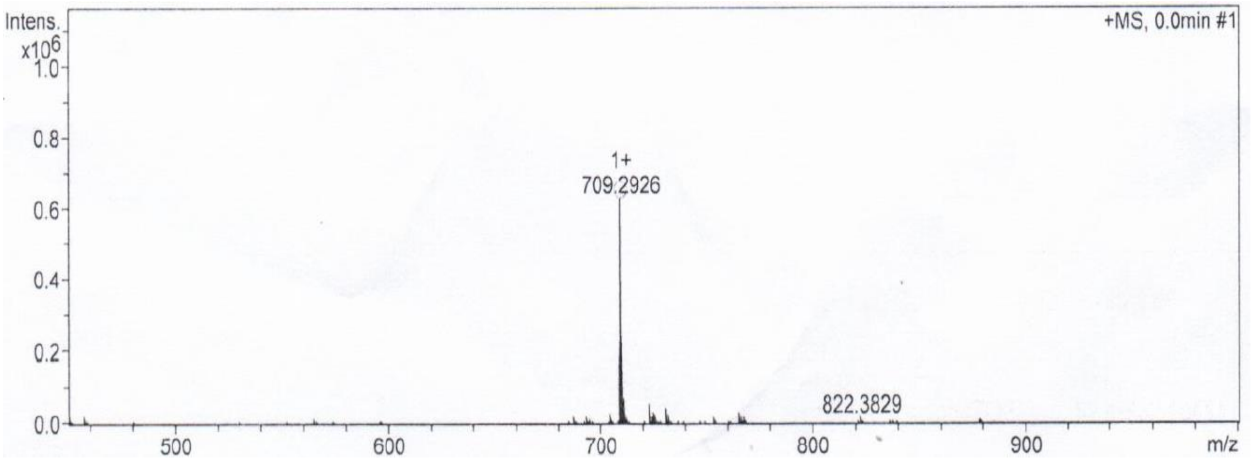

Figure S9 Mass spectrum of tetrahydrocurcumin-di-isoleucine (2c)

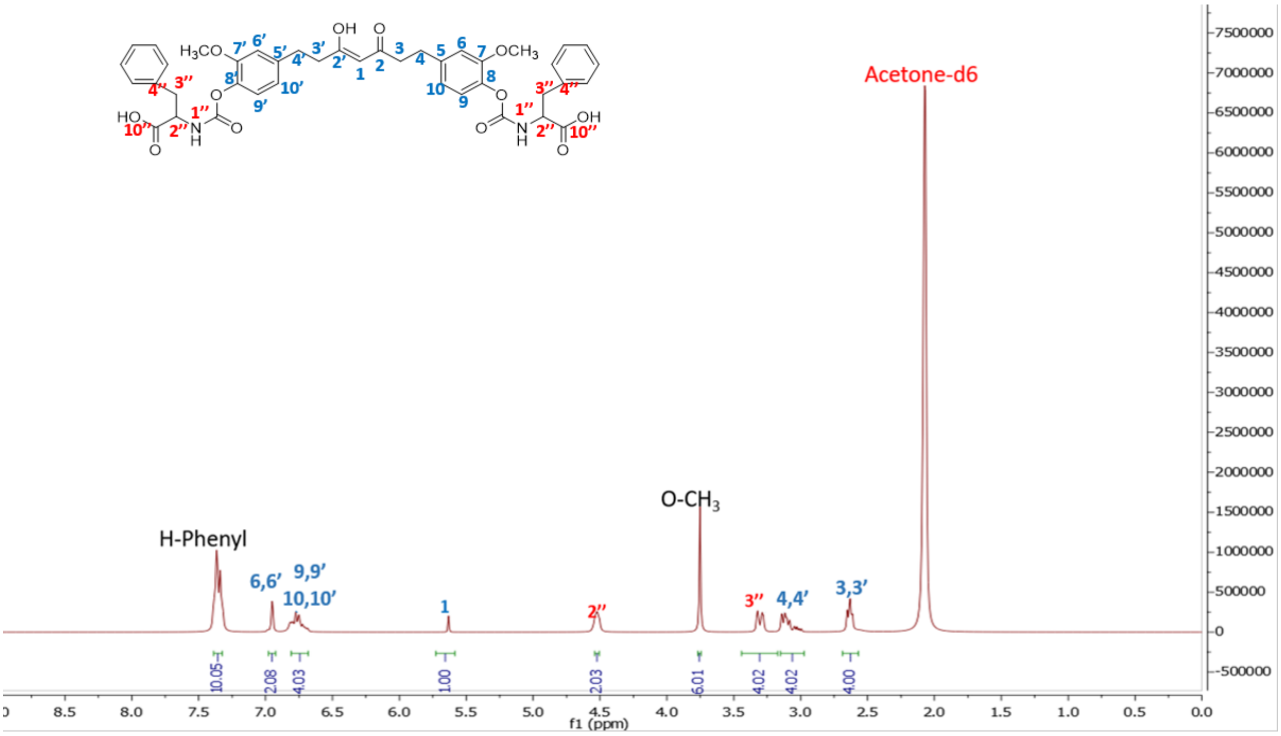

Figure S10 ^{1}H NMR spectrum of tetrahydrocurcumin-di-phenylalanine (2d)

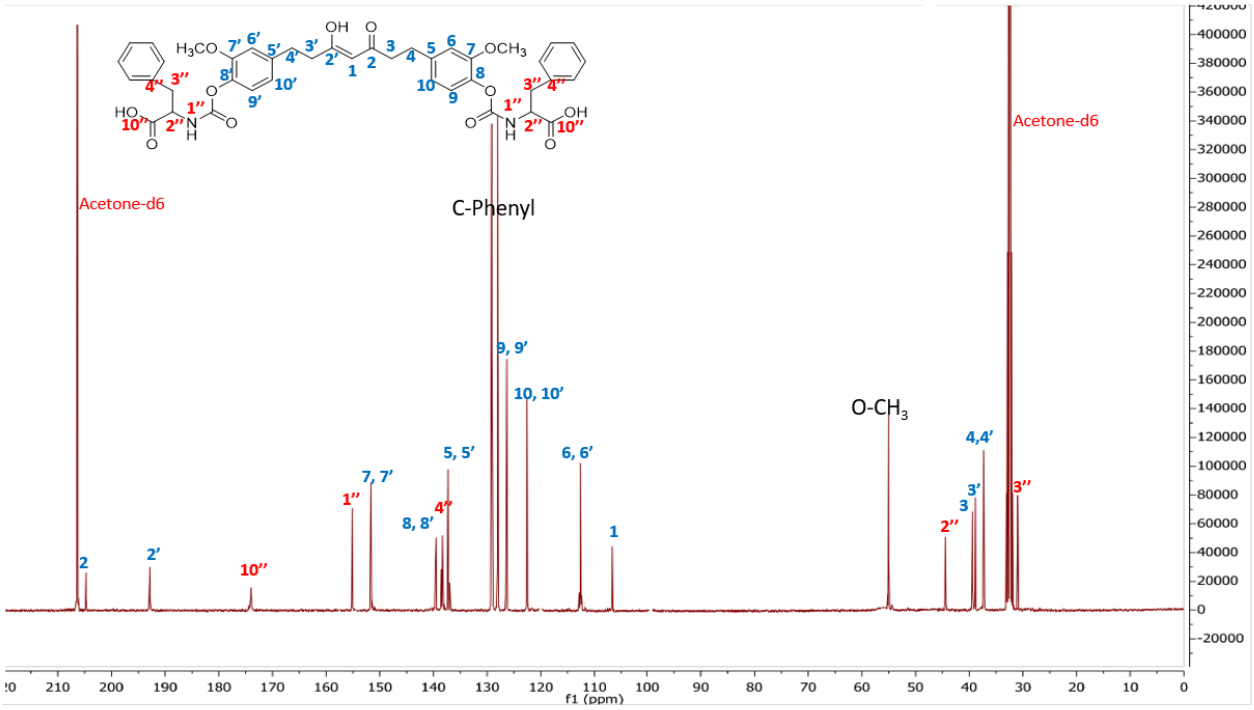

Figure S11 ^{13}C NMR spectrum of tetrahydrocurcumin-di-phenylalanine (2d)

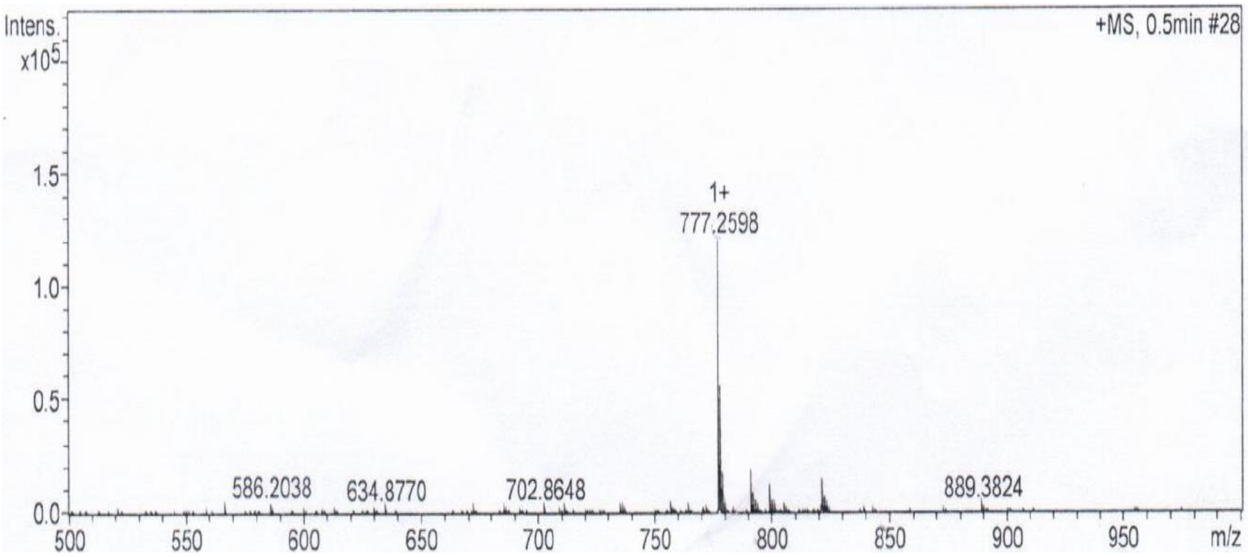

Figure S12 Mass spectrum of tetrahydrocurcumin-di-phenylalanine (2d)

Table S3 Molecular weight of compounds 2a-2d detected by HRMS.

Cpd	Structure	MW	[M+Na+] Calculated	[M+Na+] Found	Mass Accuracy (ppm)
2a	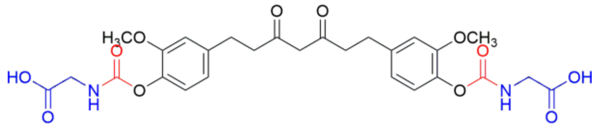	574.5390	597.1691	597.1678	-2.18
2b	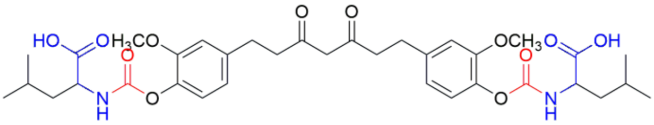	686.7550	709.2943	709.2920	-3.24
2c	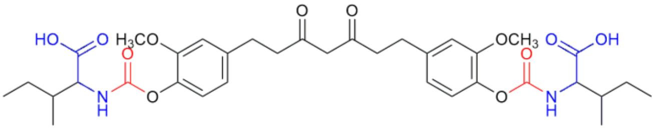	686.7550	709.2943	709.2926	-2.40
2d	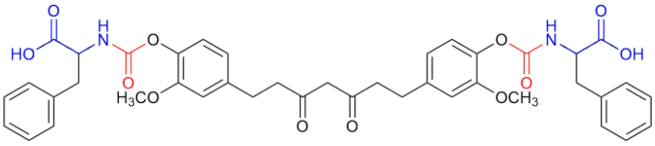	754.7890	777.2629	777.2598	-3.98

Table S4 ^{1}H-NMR (400MHz, compounds a-d in $CDCl_3$ and acetone-d6 (δ in ppm, *J* in Hz

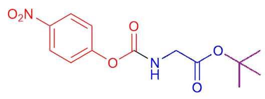

a

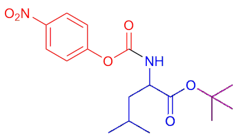

b

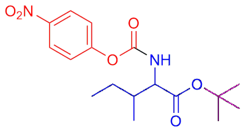

c

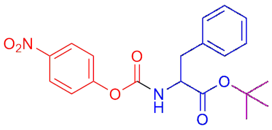

d

**Position**	**Chemical shift in ppm (multi, *J* value)**			
	**a**	**b**	**c**	**d**
Boc	1.52 s	1.51 s	1.52 s	1.43 s
NH	5.69 s	5.65 (d, 7.6)	5.75 (d, 8.7)	6.32 (d, 8.4)
1	3.98 (d, 5.3)	4.33 (d, 6.5)	4.29 (dd, 8.7,4.3)	4.40-4.49 m
2	7.34 (d, 9.2)	7.34 s (9.1)	7.35 (d, 9.1)	7.33 (d, 9.1)
3	8.26 (d, 9.2)	8.25 (d, 9.1)	8.26 (d, 9.1)	8.22-8.27 (d, 9.1)
4	-	1.65-1.88 m	1.97 m	3.06-3.12 m
5	-	1.01 (dd, 6.5, 2.0)	1.28 m	
6	-	1.65-1.88 m	0.98-1.03 m	
7	-	1.01 (dd, 6.5, 2.0)	0.98-1.03 m	
H-phenyl	-	-	-	7.24-7.29 m

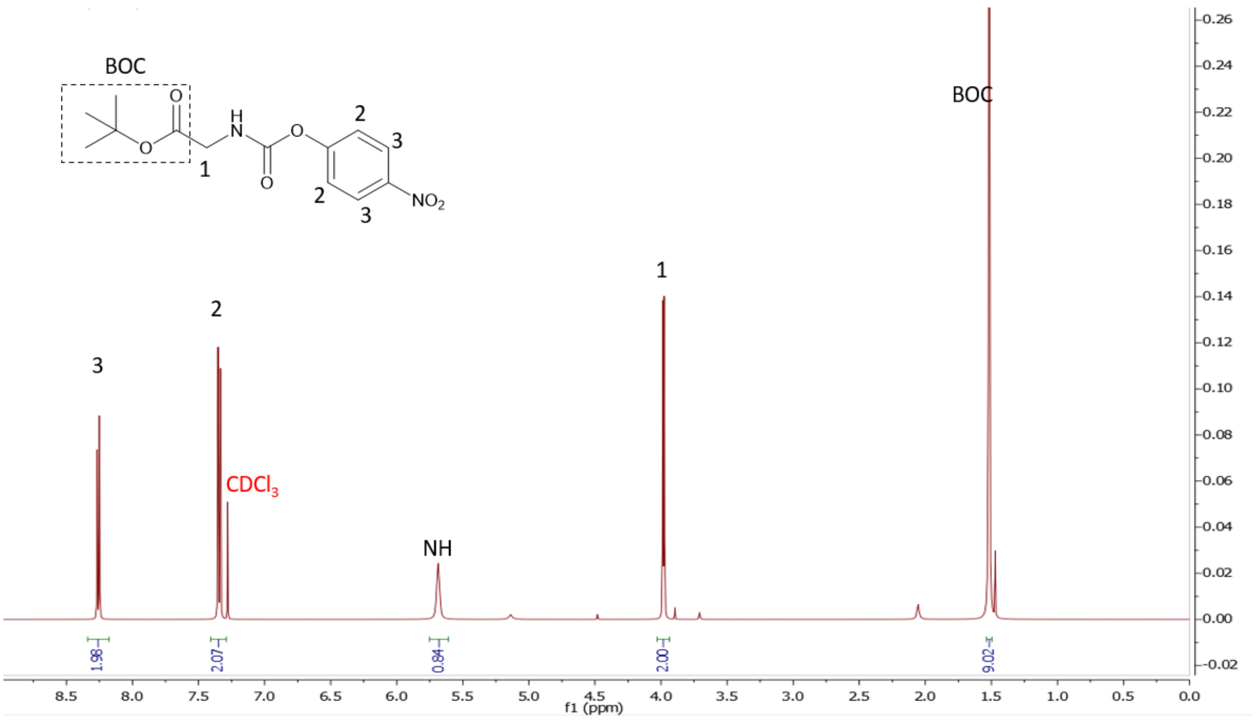

Figure S13 ^{1}H NMR spectrum of glycine activation (a)

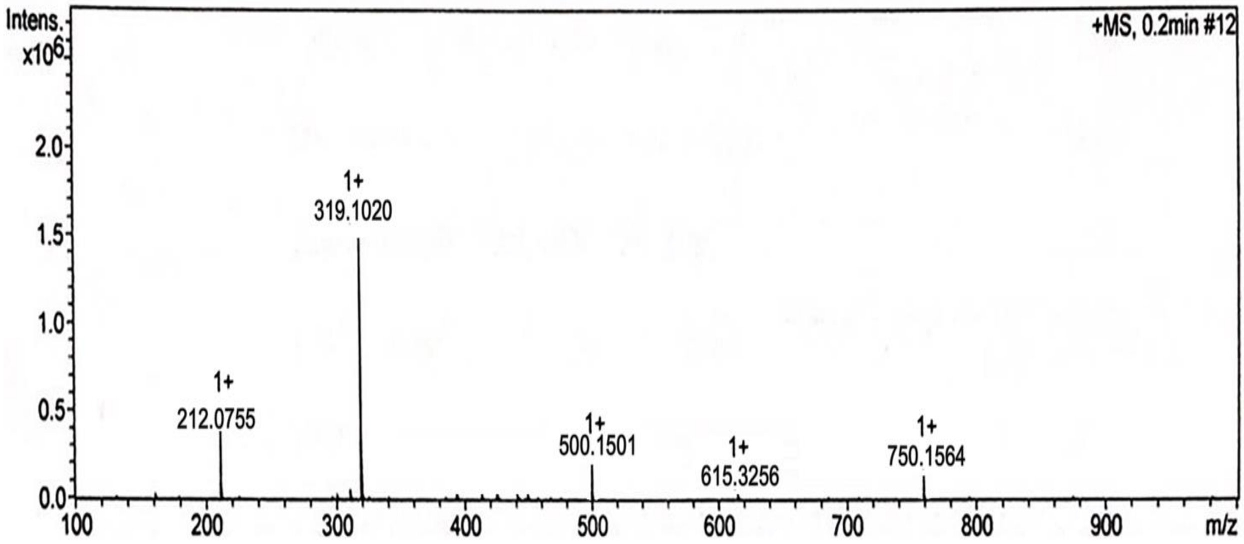

Figure S14 Mass spectrum of glycine activation (a)

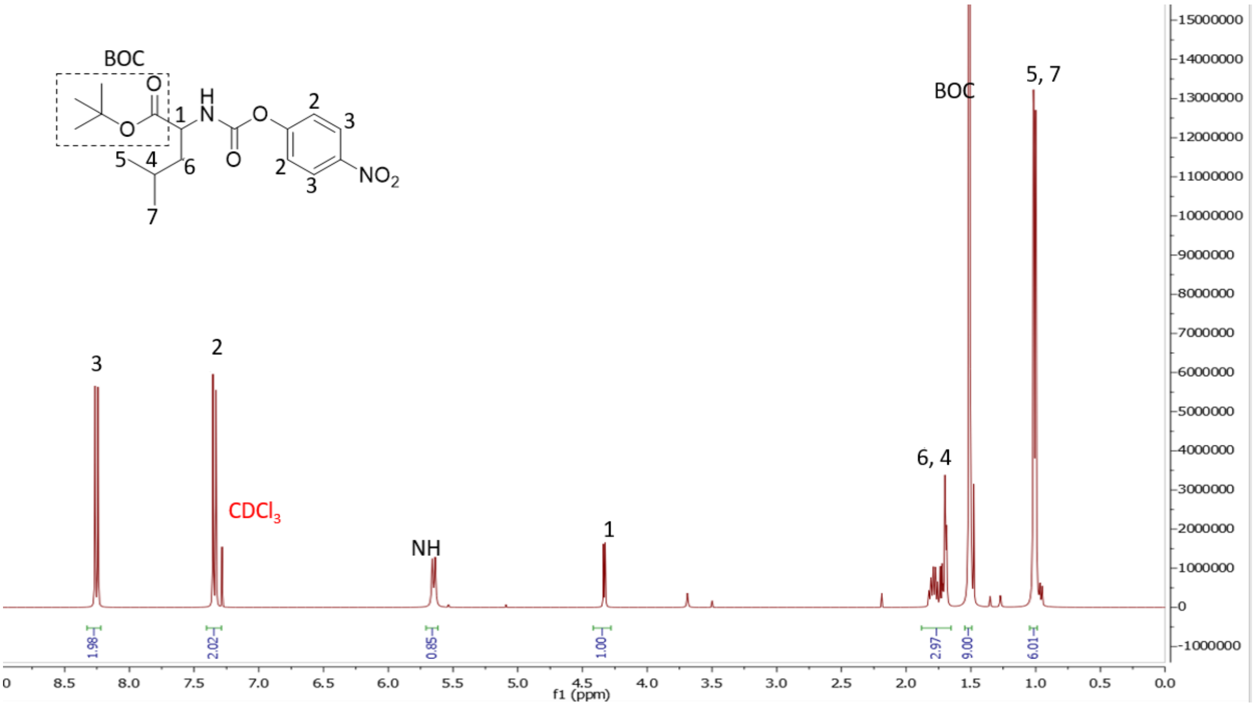

Figure S15 ^{1}H NMR spectrum of leucine activation (b)

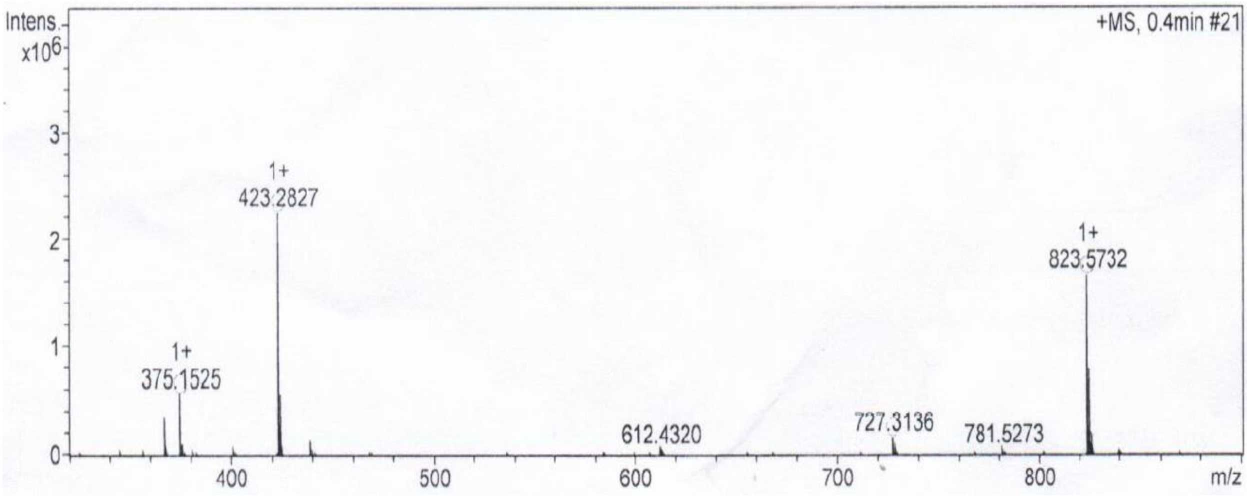

Figure S16 Mass spectrum of leucine activation (b)

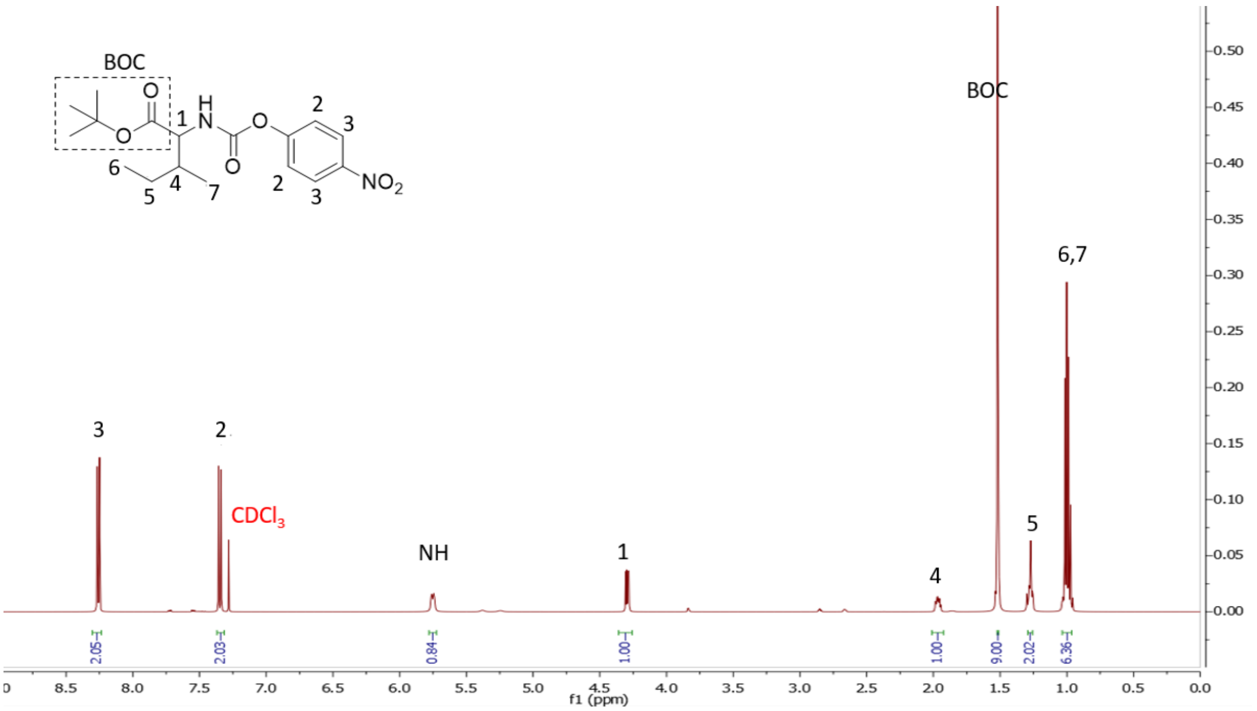

Figure S17 ^{1}H NMR spectrum of isoleucine activation (c)

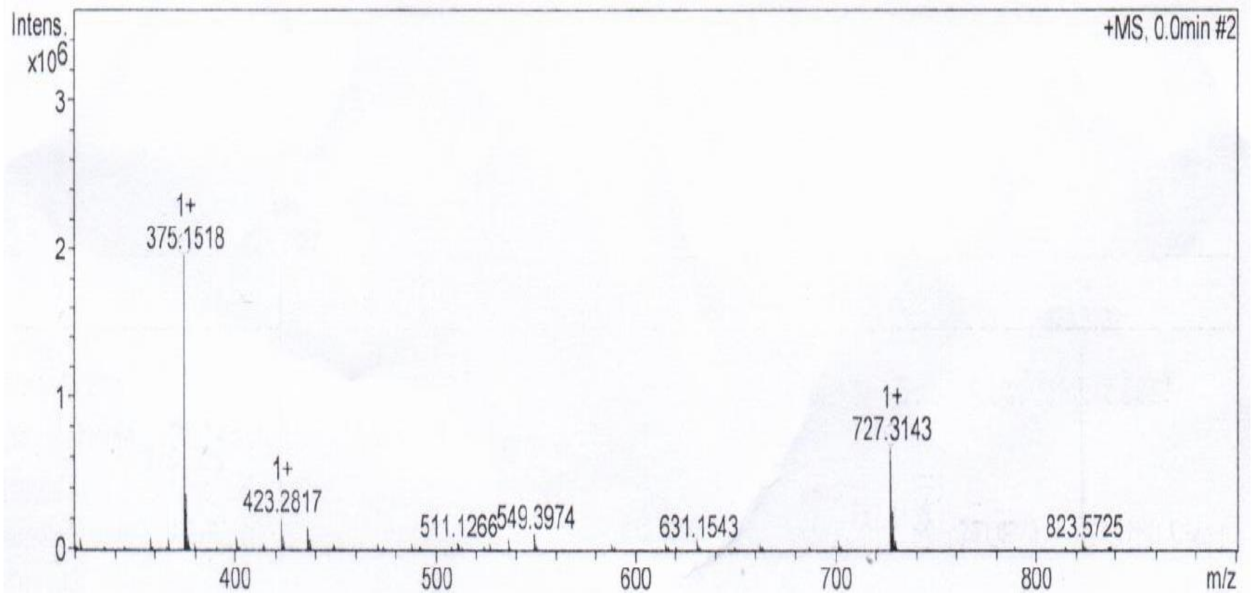

Figure S18 Mass spectrum of isoleucine activation (c)

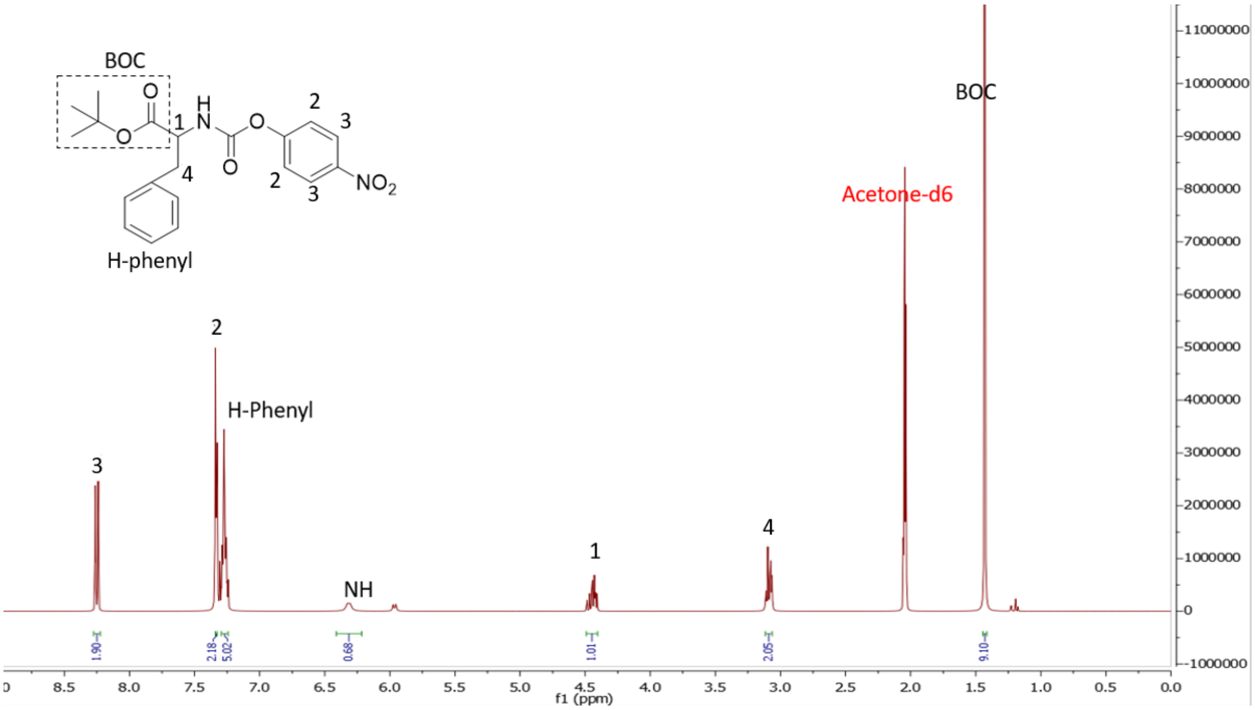

Figure S19 ^1H NMR spectrum of phenylalanine activation (d)

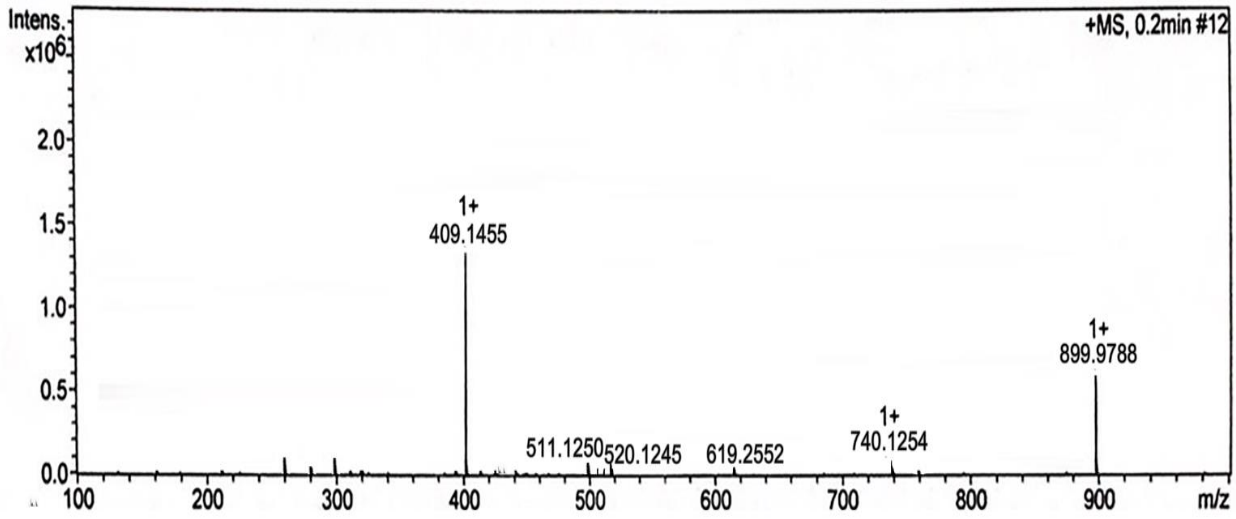

Figure S20 Mass spectrum of phenylalanine activation (d)

Table S5 Molecular weight of compounds a-d detected by HRMS.

Cpd	Structure	MW	[M+Na+]		Mass Accuracy (ppm)
			Calculated	Found	
a	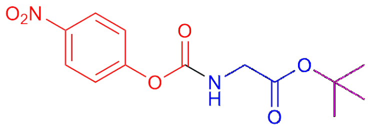	296.2790	319.1008	319.1020	3.76
b	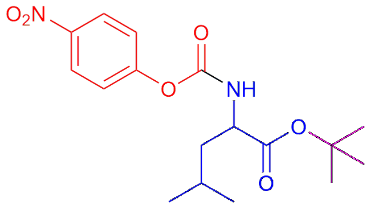	352.3870	375.1527	375.1525	-0.53
c	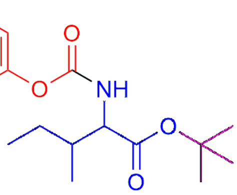	352.3870	375.1527	375.1518	-2.39
d	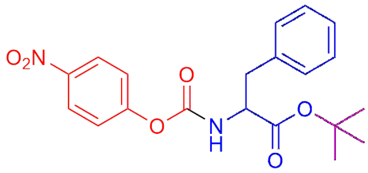	386..4040	409.1478	409.1455	-5.6

Table S6 ^{1}H-NMR (400MHz, compound 1a-1d in $CDCl_3$ and acetone-d6 (δ in ppm, *J* in Hz)

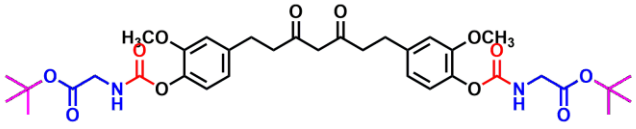

1a

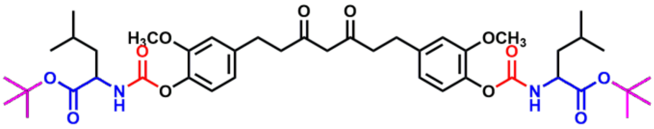

1b

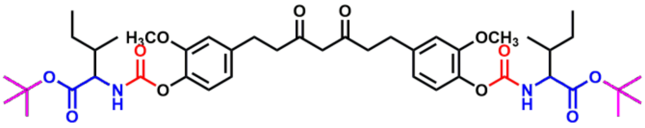

1c

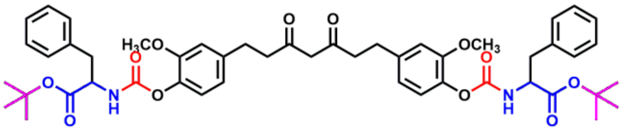

1d

**Position**	**Chemical shift in ppm (multi, *J* value)**			
	**1a**	**1b**	**1c**	**1d**
H-phenyl	-	-	-	7.26-7.29 m
Boc	1.49 s	1.50 s	1.51 s	1.44 s
O-CH_3	3.80 s	3.82 s	3.83 s	3.77 s
1	5.44 s	5.46 s	5.47 s	5.68 s
3	2.68 m	2.59 (dd, 8.9, 6.8)	2.60 (dd, 8.9, 6.8)	2.62-2.66 m
4	2.83-2.95 m	2.91 (dd, 8.9, 6.8)	2.91 (dd, 8.9, 6.8)	2.95-3.01 m
6	6.68-6.79 m	6.73-6.79 m	6.73-6.85 m	6.74-6.81 m
9	6.99 (d, 7.1)	7.01 (d, 7.9)	7.02 (d, 6.8)	6.89 (d, 6.5)
10	6.68-6.79 m	6.73-6.79 m	6.73-6.85 m	6.74-6.81 m
3′	2.56-2.59 m	2.59 (dd, 8.9, 6.8)	2.60 (dd, 8.9, 6.8)	2.62-2.66 m
4′	2.83-2.95 m	2.91 (dd, 8.9, 6.8)	2.91 (dd, 8.9, 6.8)	2.95-3.01 m
6′	6.68-6.79 m	6.73-6.79 m	6.73-6.85 m	6.74-6.81 m
9′	6.99 (d, 7.1)	7.01 (d, 7.9)	7.02 (d, 6.8)	6.89 (d, 6.5)
10′	6.68-6.79 m	6.73-6.79 m	6.73-6.85 m	6.74-6.81 m
1″	5.64-5.88 m	5.59 (d, 8.5)	5.67 (d, 8.6)	5.97 (d, 8.1)
2″	3.93 (d, 5.3)	4.30-4.34 m	4.28 (dd, 8.6,4.5)	4.51-4.53 m
3″	-	1.72-1.85 m	1.92-1.97 m	3.14-3.26 m
4″		1.72-1.85 m	1.25-1.3 m	-
5″	-	0.98-1.01 m	0.96-1.01 m	-
6″	-	0.98-1.01 m	0.96-1.01 m	-

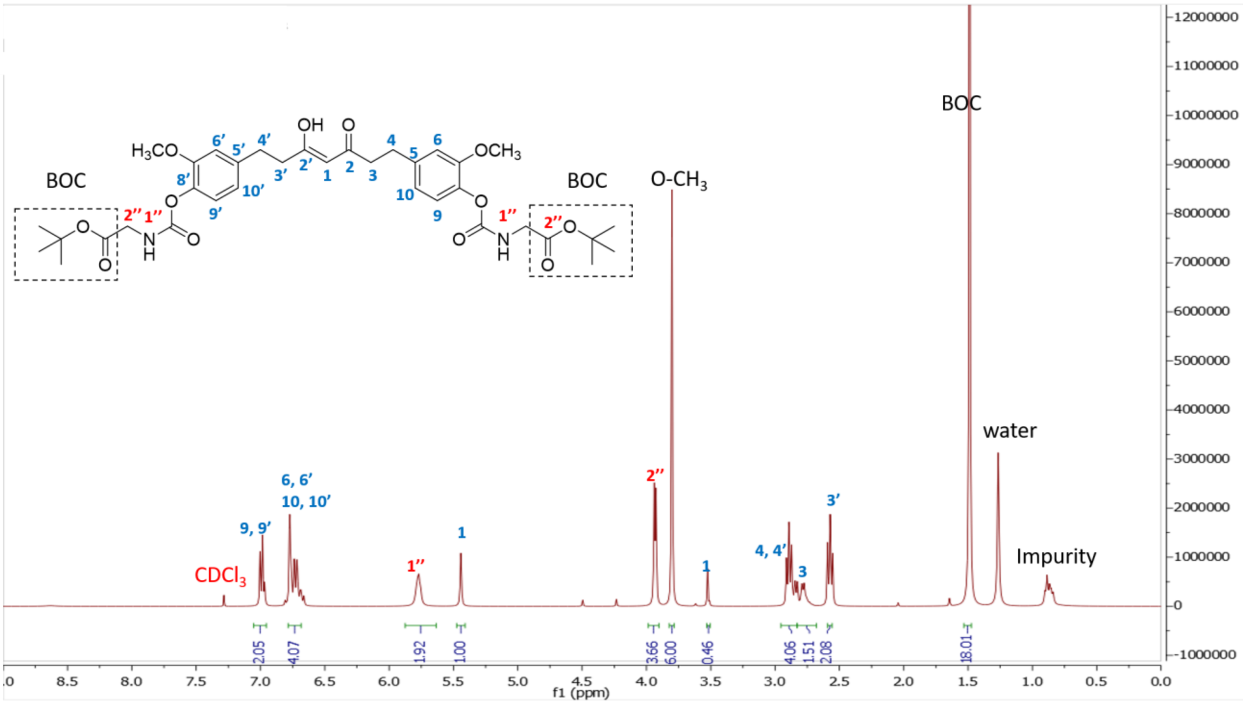

Figure S21 ^{1}H NMR spectrum of tetrahydrocurcumin-di-glycineBOC (1a)

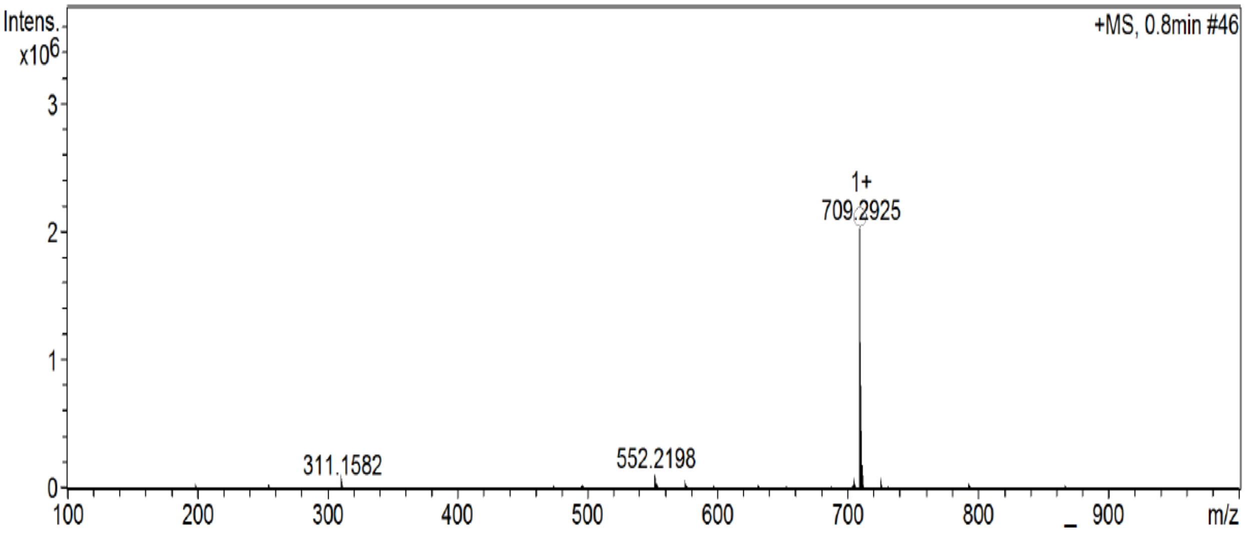

Figure S22 Mass spectrum of tetrahydrocurcumin-di-glycineBOC (1a)

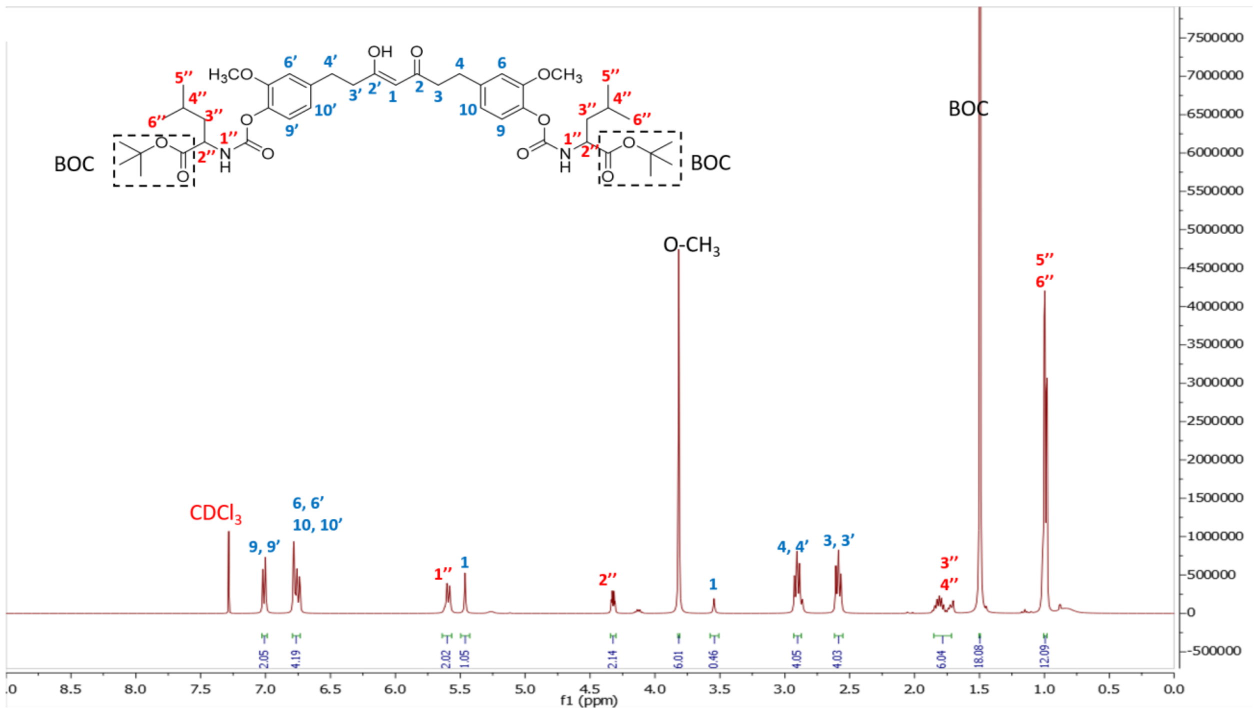

Figure S23 ^{1}H NMR spectrum of tetrahydrocurcumin-di-leucineBOC (1b)

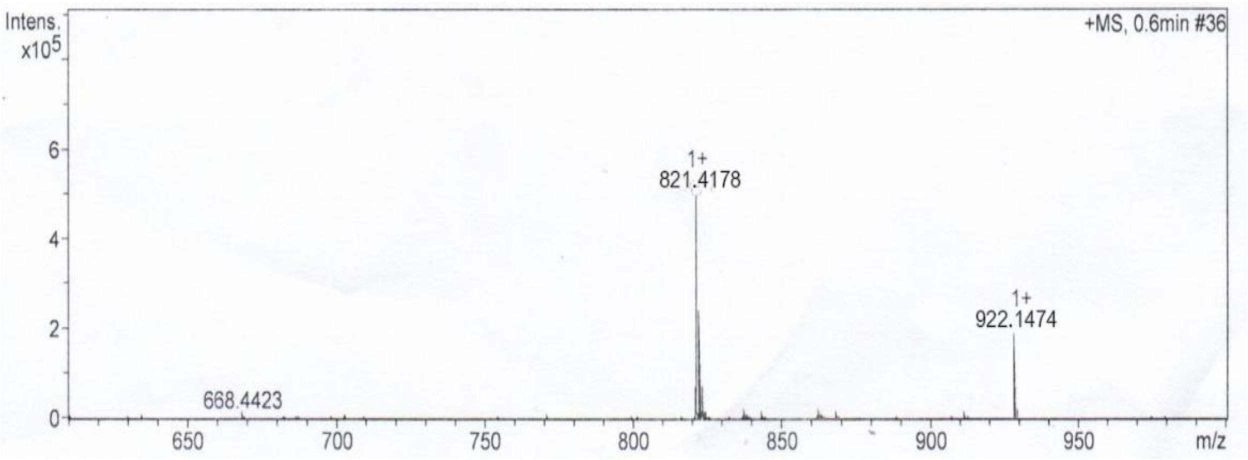

Figure S24 Mass spectrum of tetrahydrocurcumin-di-leucineBOC (1b)

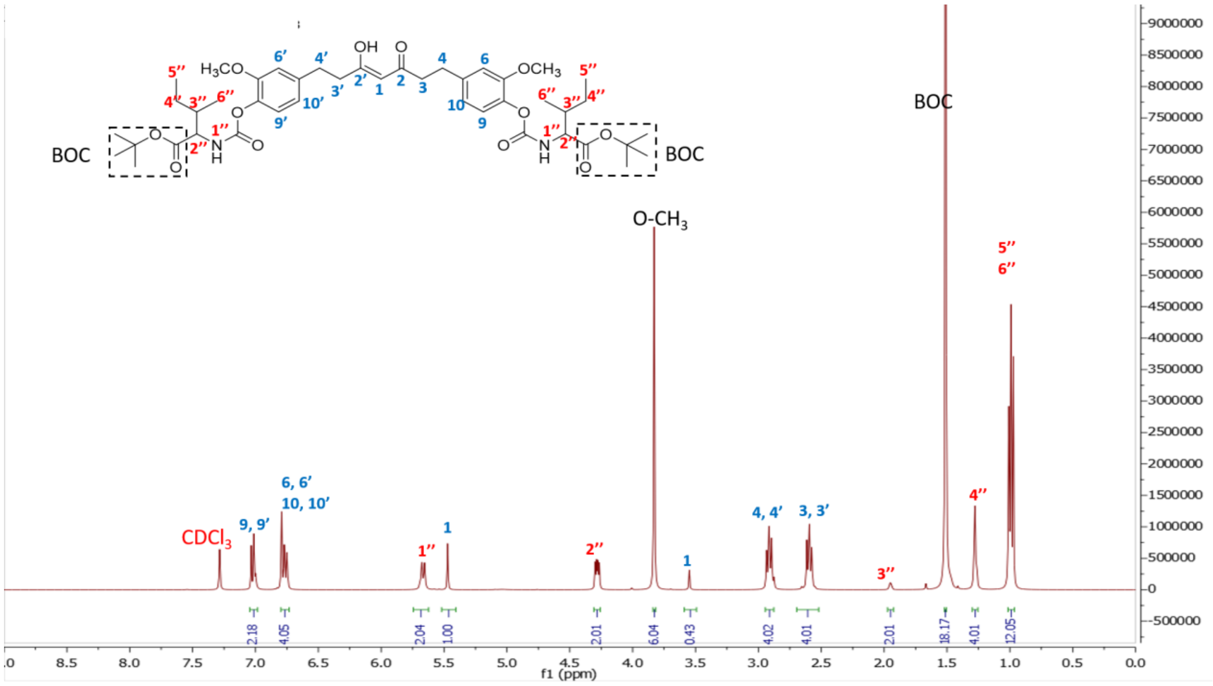

Figure S25 ^{1}H NMR spectrum of tetrahydrocurcumin-di-isoleucineBOC (1c)

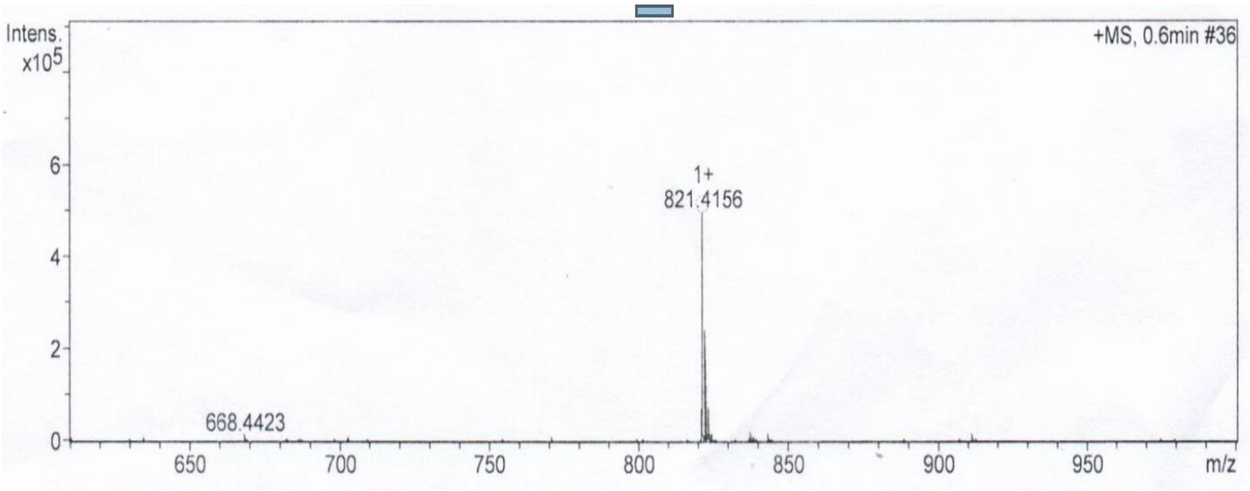

Figure S26 Mass spectrum of tetrahydrocurcumin-di-isoleucine BOC (1c)

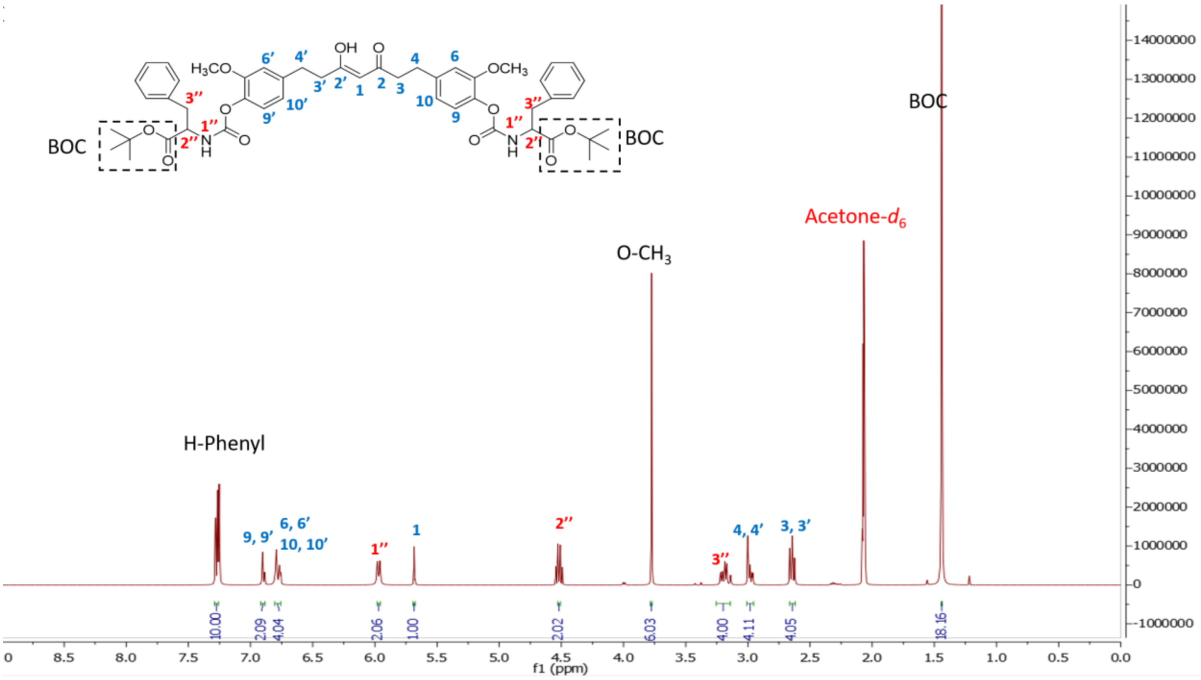

Figure S27 ^{1}H NMR spectrum of tetrahydrocurcumin-di-phenylalanineBOC (1d)

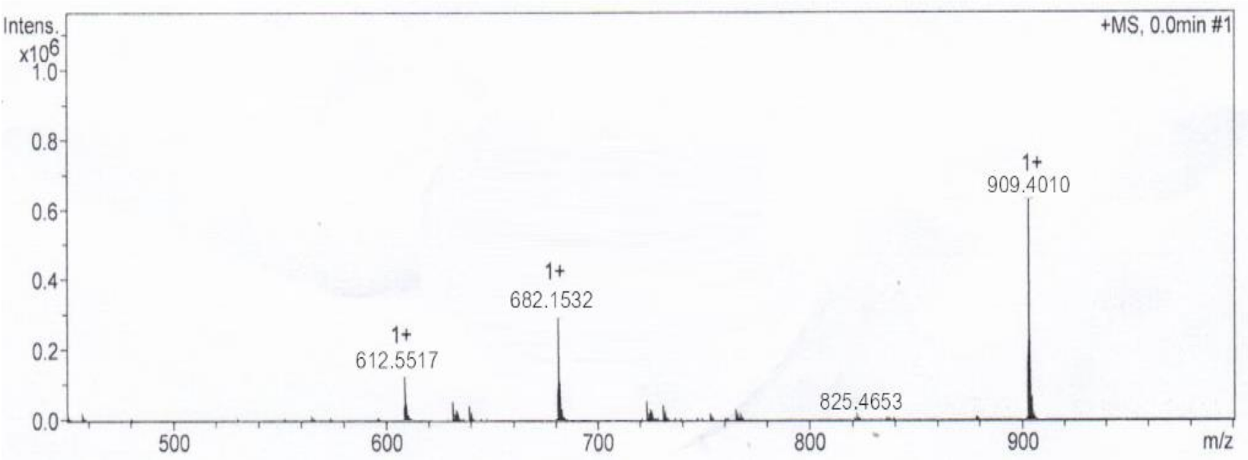

Figure S28 Mass spectrum of tetrahydrocurcumin-di-phenylalanineBOC (1d)

Table S7 Molecular weight of compounds 1a-1d detected by HRMS.

Cpd	Structure	MW	[M + Na⁺] Calculated	[M + Na⁺] Found	Mass Accuracy (ppm)
1a	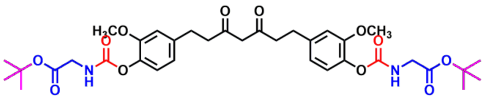	686.7550	709.2943	709.2925	-2.53
1b	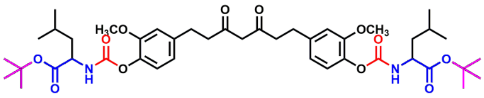	798.9710	821.4195	821.4178	-2.06
1c	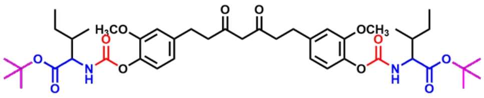	798.9710	821.4195	821.4156	-4.74
1d	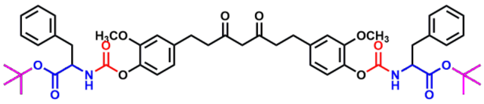	867.0050	909.3990	909.4010	2.19

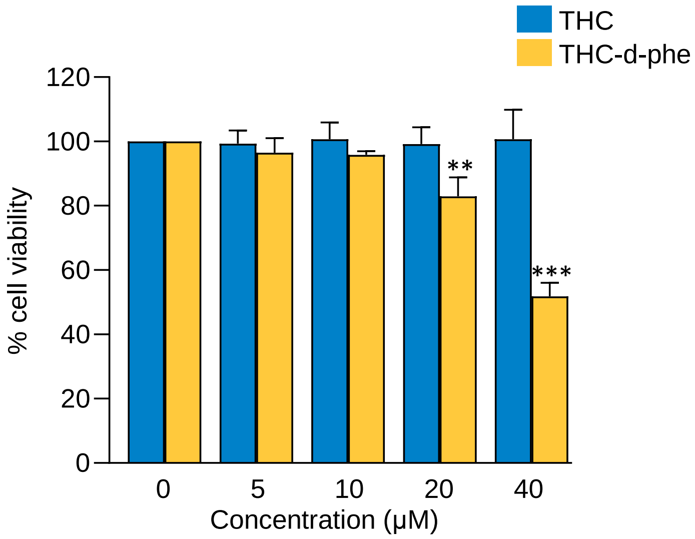

Figure S29 Cytotoxicity profiles of THC-amino acid conjugates in C6 glioma cells.

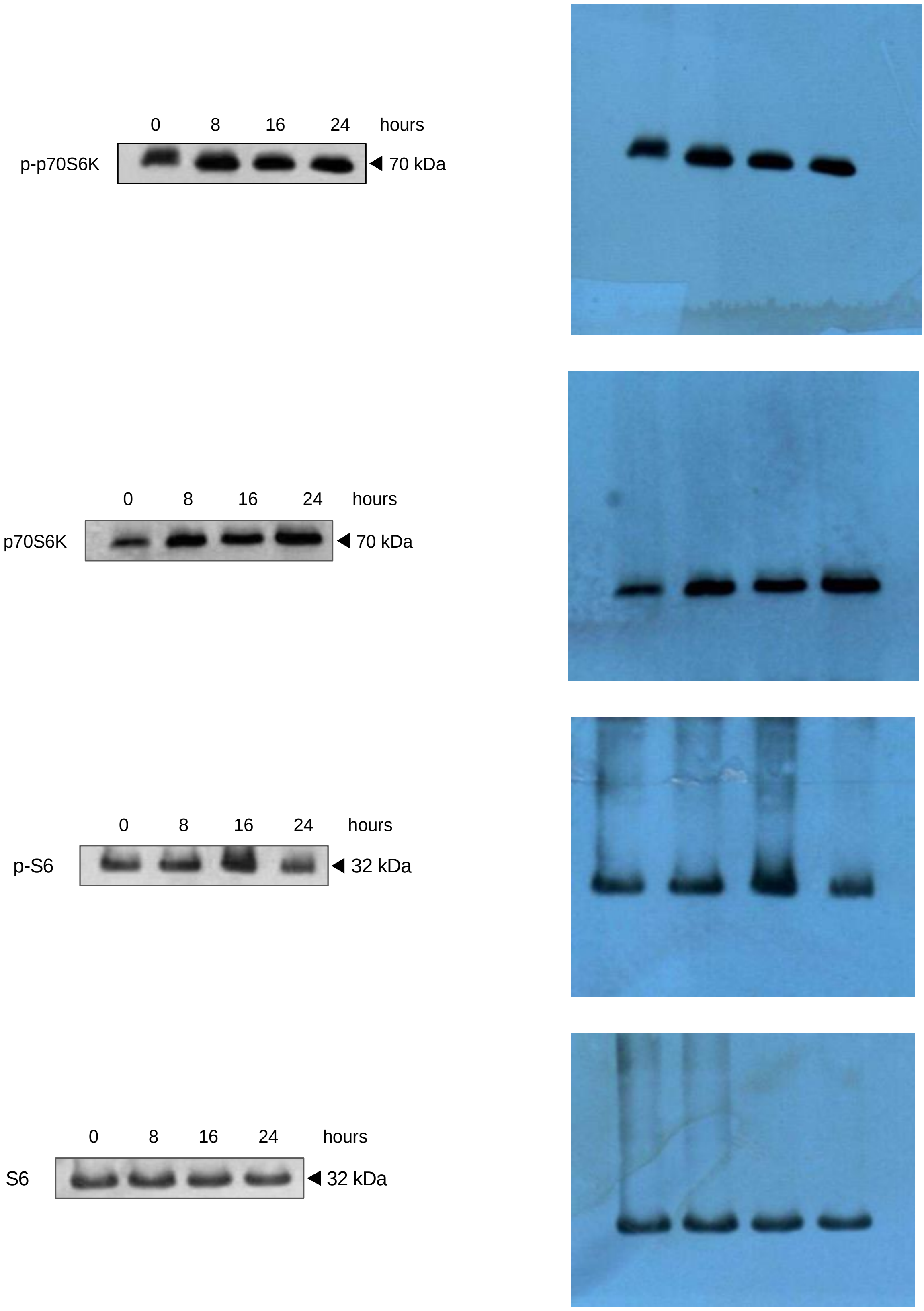

Figure S30 RAW western blot images of THC on the proteins of the P70S6K/S6 pathway at different time frames (0, 8, 16, and 24 h post-treatment).

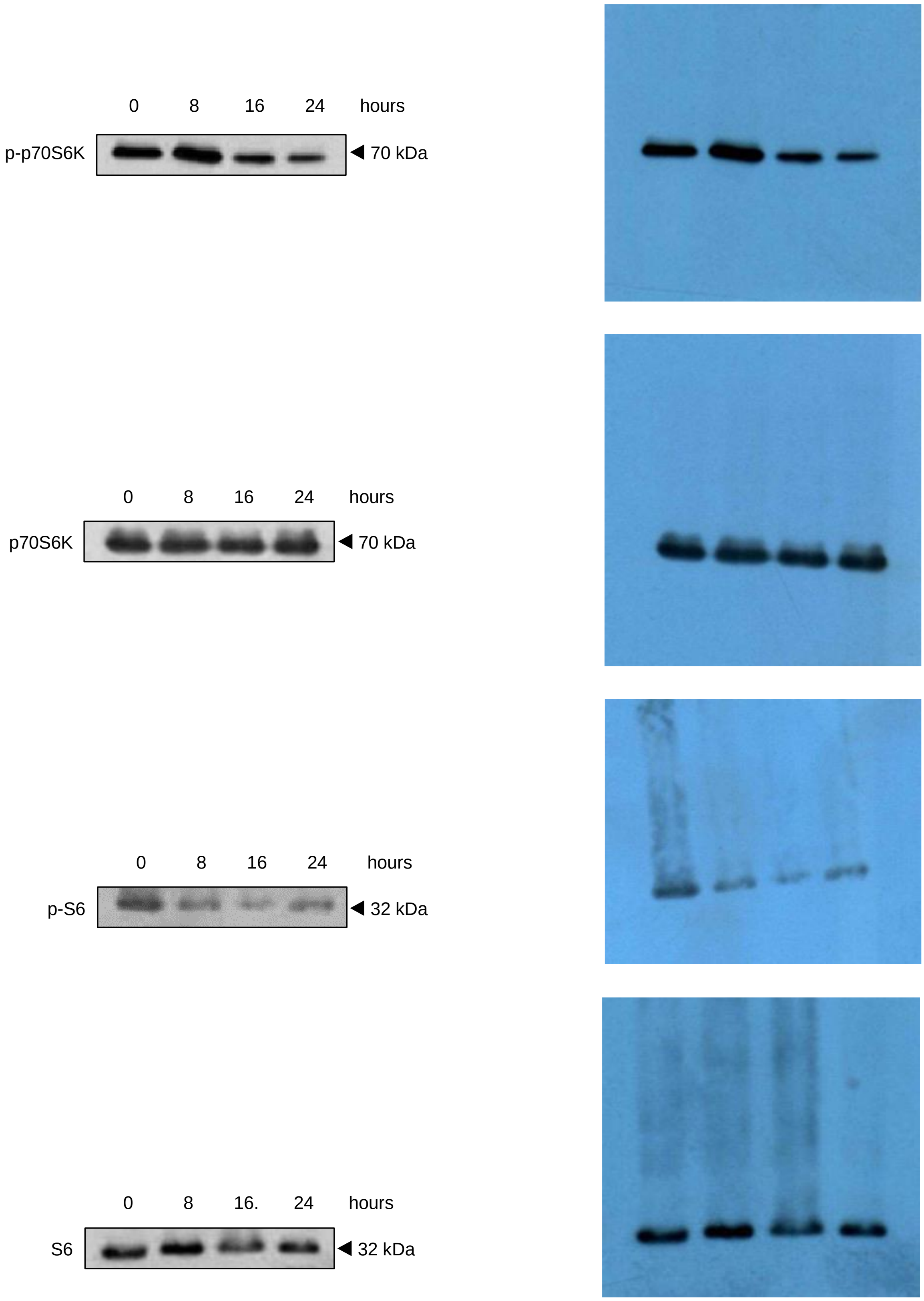

Figure S31 RAW western blot images of THC-di-Phe on the proteins of the P70S6K/S6 pathway at different time frames (0, 8, 16, and 24 h post-treatment).
